# Evaluation of *Vibrio natriegens* as a fast-growing alternative host for plasmid DNA production

**DOI:** 10.1186/s12934-026-03026-6

**Published:** 2026-05-15

**Authors:** Lara S. Möller, Gabriel A. Monteiro, Duarte M. F. Prazeres, A. Rita Silva-Santos

**Affiliations:** 1 iBB – Institute for Bioengineering and Biosciences, Department of Bioengineering, Instituto Superior Técnico, Universidade de Lisboa, Av. Rovisco Pais 1, 1049-001 Lisbon, Portugal; 2 Associate Laboratory i4HB – Institute for Health and Bioeconomy, Instituto Superior Técnico, Universidade de Lisboa, 1049-001 Lisbon, Portugal; 3 Hochschule Bremen City University of Applied Sciences, Neustadtswall 30, 28199 Bremen, Germany

**Keywords:** Bioprocess intensification, *Escherichia coli*, Microbial cell factory, Plasmid DNA production, Rapid biomanufacturing, *Vibrio natriegens*, Volumetric productivity

## Abstract

**Background:**

Plasmid DNA (pDNA) is a key biomolecule for gene therapies, DNA vaccines, and mRNA-based applications. Although *Escherichia coli* is the established host for pDNA production, limitations such as moderate growth rates and endotoxin-associated challenges motivate the exploration of alternative production hosts. *Vibrio natriegens* is a fast-growing, non-pathogenic bacterium with a doubling time below 10 min and high metabolic versatility. While it has been adopted for cloning and protein expression, its potential for pDNA production remains largely unexplored.

**Results:**

We evaluated pDNA production by *V. natriegens* in comparison to *E. coli* in shake flask cultivations across four complex media (LB, BHI, 2xYT, TB) and one defined minimal medium (MSM). Under all tested conditions, *V. natriegens* exhibited faster biomass accumulation and enabled earlier pDNA isolation. Quantitative analysis of triplicate experiments revealed that *V. natriegens* achieved comparable or higher volumetric and specific pDNA yields in shorter cultivation times than *E. coli*. Agarose gel electrophoresis confirmed plasmid integrity, and functional validation using a cell-free expression system demonstrated efficient eGFP reporter expression from *V. natriegens*-derived plasmids.

**Conclusions:**

This proof-of-concept study demonstrates that *V. natriegens* is a promising alternative host for pDNA production. Its combination of accelerated growth, robust pDNA yields, and functional plasmid quality highlights its potential for further development as a pDNA production platform.

**Supplementary Information:**

The online version contains supplementary material available at 10.1186/s12934-026-03026-6.

## Introduction

Plasmid DNA (pDNA) is a central biomolecule in biotechnology and medicine, serving as a versatile genetic vector for gene therapies, DNA vaccines, and as a template for in vitro-transcribed mRNA [1, 2]. Due to its chemical stability and circular double-stranded structure, pDNA enables cost-efficient manufacturing and long-term storage, which are critical for biomedical and industrial applications [3, 4]. At the same time, stringent quality requirements must be fulfilled, including maintenance of the supercoiled plasmid topology and effective removal of host-derived impurities such as genomic DNA and endotoxins, particularly under Good Manufacturing Practice (GMP) conditions [3, 5].

Over the past decades, *Escherichia coli* has become the dominant host for industrial pDNA production due to its well-characterized genetics, high plasmid copy numbers, and established fermentation strategies [1, 2]. Laboratory strains such as *E. coli* DH5α harbor mutations that improve plasmid stability during cloning by reducing recombination and nuclease activity, but they are not optimized for large-scale manufacturing [2]. Such large-scale pDNA production is typically performed under fed-batch conditions, where controlled growth rates, substrate limitation, and process optimization are used to enhance plasmid yield, stability, and process robustness [5, 6]. Nevertheless, DH5α represents a widely used and well-characterized laboratory strain that provides a consistent and reproducible reference for comparative evaluation under standardized batch conditions, rather than serving as a proxy for optimized large-scale production strains.

However, several limitations of *E. coli* have been described. As a Gram-negative organism, *E. coli* produces lipopolysaccharides (LPS), which necessitate extensive downstream purification to meet regulatory requirements for pharmaceutical applications [3]. In addition, plasmid replication imposes a metabolic burden that can impair host fitness and productivity, particularly under high-growth conditions. Overflow metabolism and acetate accumulation may further constrain efficient pDNA production and process robustness [7–9].

To address these limitations, alternative microbial hosts have been explored. Gram-positive organisms such as *Lactococcus lactis* offer endotoxin-free production platforms and have been investigated for pharmaceutical DNA manufacturing; however, their comparatively slow growth rates and lower plasmid copy numbers limit scalability and productivity [10, 11]. As a result, there is growing interest in fast-growing and metabolically flexible organisms, especially for workflows that benefit from rapid biomass accumulation and short turnaround times, such as early-stage research, cloning, and screening [12].

In this context, *Vibrio natriegens* has emerged as a promising candidate for such applications. As a Gram-negative organism, *V. natriegens* also produces lipopolysaccharides (LPS), which represent a key consideration for biopharmaceutical applications. However, structural differences in the lipid A and inner core regions, particularly involving Kdo phosphorylation, have been reported for *Vibrio* species and are known to influence endotoxin activity [13–15]. These modifications can affect interactions with host factors and may result in reduced endotoxic activity compared to *E. coli* [13, 14]. Nevertheless, endotoxin removal remains a necessary step in downstream processing for both organisms, although differences in endotoxin activity may influence the extent of purification required.

Originally isolated from marine environments, *V. natriegens* exhibits the fastest known doubling time among free-living organisms, with reported values below 10 min under optimal conditions [16]. This exceptional growth performance has been attributed to its high ribosomal RNA operon copy number and efficient nutrient uptake systems [17, 18].

Recent years have seen substantial progress in the development of molecular and synthetic biology tools for *V. natriegens*, including compatible plasmid backbones, CRISPR-based regulation systems, and efficient genome editing strategies such as Multiplex Genome Editing by Natural Transformation (MuGENT) [19–21]. Engineered strains have demonstrated robust recombinant protein expression and broad metabolic flexibility, supporting their application as alternative biotechnological chassis [22–25]. Despite these advances, the potential of *V. natriegens* as a host for plasmid DNA production has received comparatively little attention, and systematic evaluations in direct comparison with *E. coli* remain limited.

The *V. natriegens* Vmax strain used in this study is an engineered derivative carrying a deletion of dns, which encodes an extracellular, nonspecific endonuclease, according to supplier specifications (Telesis Bio). This modification parallels the classical endA⁻ strategy implemented in *E. coli* strains and reduces nuclease activity, thereby limiting plasmid degradation during cell disruption and downstream processing.

For comparison, *E. coli* K-12 DH5α, a widely used cloning strain carrying mutations that reduce recombination (recA1) and nuclease activity (endA1), thereby improving plasmid stability and yield, was selected as a reference host [26]. While these genetic features differ in their specific functions, both strains are optimized to support plasmid maintenance and recovery, providing a suitable basis for comparative evaluation.

In this study, we present a proof-of-concept evaluation of *V. natriegens* as an alternative host for plasmid DNA production. We systematically compare growth behavior and volumetric pDNA yields of *V. natriegens* and *E. coli* in shake flask cultivations across four complex media and one defined minimal medium. In addition, plasmid integrity and functional performance are assessed using agarose gel electrophoresis and a cell-free expression system. By directly benchmarking *V. natriegens* against the established *E. coli* cloning and production host under standardized conditions, this work aims to provide a foundational assessment of its suitability for rapid and efficient pDNA production under batch cultivation conditions, with a focus on time-sensitive and small-scale applications.

## Materials and methods

Unless otherwise specified, all chemicals were purchased from Sigma-Aldrich (USA).

### Bacterial strains and plasmid

*Vibrio natriegens* Vmax (Vmax X2, Telesis Bio, USA) and *Escherichia coli* DH5α (Thermo Fisher Scientific, USA) were used as host strains for pDNA production.

The plasmid peGFP (2967 bp; Supplementary Fig. S1), carrying an ampicillin resistance marker and encoding enhanced green fluorescent protein (eGFP) under control of a T7 promoter, was used as the primary model construct in this study. The plasmid was constructed in-house and contains a pBR322-derived ColE1 origin of replication for propagation in *E. coli*. The final construct was verified by Sanger sequencing.

To assess the applicability of *V.natriegens* Vmax for plasmid production across different vector backbones, additional high-copy plasmids were analyzed (Supplementary Fig. S1), including pCEP4 (10,410 bp; Invitrogen, USA), pUC18 (2686 bp; Thermo Fisher Scientific, USA), and pUC57 (3882 bp; NZYTech, Portugal). All plasmids carried an ampicillin resistance marker and were propagated in the respective host strains prior to cultivation experiments.

### Transformation and cell bank preparation

Chemically competent cells of *V. natriegens* Vmax were prepared based on the protocol described by [25]. Cells were cultivated in LB medium supplemented with V2 salts (204 mM NaCl, 4.2 mM KCl, 23.1 mM MgCl_2_ [27]), at 30 °C with shaking at 120 rpm until OD_600_ ≈ 0.5. Cells were harvested by centrifugation at 4 °C, 5000 × g for 10 min and rendered competent using CCMB80 buffer (10 mM potassium acetate, 80 mM CaCl_2_·2H_2_O, 20 mM MnCl_2_·2H_2_O, 10 mM MgCl_2_·6H_2_O, 25% (v/v) glycerol, pH 6.4). Aliquots were stored at − 80 °C until use.

Transformation of *V. natriegens* Vmax was performed by heat shock for 1 min at 42 °C followed by incubation with pDNA on ice for 3 min. After recovery in LB(+ V2) medium for 2 h at 37 °C with shaking, transformants were selected on LB(+ V2) agar plates containing ampicillin (100 µg mL⁻¹).

Competent *E. coli* DH5α cells were prepared using the TSS method as described by [28]. Cells were grown in LB medium at 37 °C with shaking at 250 rpm until OD_600_ ≈ 0.4 and rendered competent using TSS buffer (LB supplemented with 10% (w/v) PEG, 5% (v/v) DMSO, 50 mM MgCl_2_, pH 6.5). Aliquots were stored at − 80 °C. Transformation was performed by heat shock for 45 s at 42 °C, followed by a 2 min incubation on ice. Recovery followed in LB medium for 1 h at 37 °C and selection on LB agar plates containing ampicillin (100 µg mL⁻¹).

### Media and cultivation conditions

Shake flask cultivations were performed in four complex media (LB, BHI, 2xYT, and TB) and in a defined mineral salt medium (MSM [29], Supplementary Tab. S2.). For *V. natriegens* Vmax, all media were supplemented with V2 salts [27]. Ampicillin (100 µg mL⁻¹) was added to all cultures to ensure plasmid maintenance.

Pre-cultures were inoculated from glycerol stocks (− 80 °C) and grown overnight in 30 mL of the respective medium at 37 °C with shaking at 250 rpm for both *V. natriegens* Vmax and *E. coli* DH5α. A temperature of 37 °C, is within the reported growth range for *V. natriegens*, was selected to ensure direct comparability between both host organisms under identical cultivation conditions. For MSM cultures, pre-cultures were grown in 2xYT medium. Production cultures were inoculated to an initial OD_600_ of 0.1 into 30 mL medium in 250 mL baffled shake flasks and incubated at 37 °C with shaking at 250 rpm.

Cultivations in complex media were performed for 6 h, while cultures grown in MSM were extended to 8 h to account for the longer lag phase observed under defined medium conditions. Biomass formation was monitored by measuring OD_600_ at hourly intervals throughout the cultivation, using identical sampling intervals for both strains and all media.

Biomass concentrations were calculated as dry cell weight (DCW) using experimentally determined, strain-specific OD_600_-to-DCW conversion factors (L-factor, L) according to:

$$\:Biomass\:\left[g\:{L}^{-1}\right]={OD}_{600}\times\:L$$

L-factors of 0.45 and 0.50 g DCW L⁻¹ OD_600_⁻¹ were applied to *V. natriegens* Vmax and *E. coli* DH5α, respectively.

### Plasmid DNA extraction and quantification

pDNA was extracted from shake flask cultures at defined time points using the High Pure Plasmid Isolation Kit (Roche, Switzerland) according to the manufacturer’s instructions. For each extraction, 2 mL of culture was sampled and diluted on ice to an optical density of OD_600_ = 1.0 to ensure comparable processing conditions while minimizing metabolic activity during sample handling. The diluted samples were centrifuged (3 min, 10,000 × g, 4 °C), and the resulting pellets were stored at − 20 °C until further processing.

Samples with OD_600_ values below 1.0, which occurred predominantly in early cultivation phases of *E. coli* DH5α, were not included in the quantitative pDNA yield analysis, as they were below the recommended input range and detection limit of the extraction kit, resulting in insufficient comparability across strains and time points.

For qualitative assessment of plasmid presence and integrity, pDNA was also extracted from selected early time points (e.g., 2 h), particularly from *E. coli* DH5α cultures that did not meet the OD_600_ threshold for quantitative analysis. These samples were analyzed by agarose gel electrophoresis and to assess topology and integrity only, and were not considered for yield calculations.

pDNA was eluted in 50 µL nuclease-free water. DNA concentration and purity were determined by UV–Vis spectrophotometry using a NanoDrop One instrument (Thermo Fisher Scientific, USA). Volumetric pDNA yields (mg L⁻¹ culture) were calculated according to the following equation:

$$ Volumetric~pDNA~yield~\left[ {g~L^{{ - 1}} } \right] = \frac{{c \times V_{{elution}} \times 10^{{ - 3}} \times OD_{{culture}} }}{{V_{{sample}} }} $$

where c is the measured DNA concentration (ng µL⁻¹), V_elution_ is the elution volume (µL), OD_culture_ is the optical density (600 nm) of the culture prior to dilution, and V_sample_ is the sampled culture volume (mL).

Biomass concentrations (g L⁻¹) were calculated as described above using strain-specific OD_600_-to-DCW conversion factors.

Specific pDNA yields (mg g⁻¹ CDW) were calculated by normalizing volumetric yields to biomass concentration:

$$ Specific~pDNA~yield~\left[ {mg~g^{{ - 1}} ~DCW} \right] = \frac{{Volumetric~pDNA~yield~\left[ {mg~L^{{ - 1}} } \right]}}{{Biomass~\left[ {g~L^{{ - 1}} } \right]}} $$

### Plasmid integrity analysis

Plasmid integrity was assessed by agarose gel electrophoresis. pDNA samples (10 µL) were mixed with 6× DNA loading dye (Gel Loading Dye Purple, New England Biolabs, USA) and separated on 1% (w/v) agarose gels in 1× TAE buffer at 90 V for 1 h. A DNA size marker (NZYDNA Ladder III, 200–10,000 bp; NZYtech, Portugal) was used for band size estimation. DNA bands were visualized under UV illumination following ethidium bromide staining using a gel documentation system (Axygen, USA).

### Functional validation of plasmid DNA

The functionality of isolated pDNA was evaluated using a commercial cell-free protein expression system (NEBExpress Cell-Free *E. coli* Protein Expression System, New England Biolabs, USA), which provides the necessary transcriptional machinery, including T7 RNA polymerase. Cell-free reactions (50 µL) were prepared according to the manufacturer’s instructions using 250 ng pDNA as template. Plasmid samples isolated from three independent 2xYT medium, 6 h-shake flask cultivations per strain were analyzed. A positive control provided with the kit (DHFR template) and a no-template control were included to verify system performance and exclude background signals. Reactions were incubated at 37 °C for 4 h shaking at 250 rpm.

### Protein analysis by SDS–PAGE and western blotting

For SDS–PAGE analysis, 2 µL of each cell-free expression reaction was diluted with nuclease-free water, mixed 1:1 with 2× Laemmli sample buffer containing 50 mM DTT, and heated to 95 °C for 5 min. Proteins were separated on polyacrylamide gels consisting of a 12% resolving gel and a 5% stacking gel using a Mini-PROTEAN Tetra Cell electrophoresis system (Bio-Rad Laboratories, USA). After electrophoresis, gels were stained with Coomassie Brilliant Blue R-250 and destained until a clear background was obtained.

For Western blot analysis, proteins were transferred onto nitrocellulose membranes using a semi-dry blotting system (Power Blotter, Invitrogen, Thermo Fisher Scientific, USA). Membranes were blocked for 1 h at room temperature in blocking buffer (PBS 1×, 0.1% Tween-20, 3% (w/v) milk) and incubated overnight at 4 °C with a primary anti-GFP antibody (clone B-2, SC-9996, Frilabo, France; 1:200 dilution) diluted in antibody incubation buffer (PBS 1×, 0.1% Tween-20, 1% (w/v) milk). After washing three times for 10 min in washing buffer (PBS 1×, 0.1% Tween-20), membranes were incubated with an HRP-conjugated secondary antibody (m-IgG BP-HRP, Frilabo, France; 1:10,000 dilution) for 90 min at room temperature. Following three additional washing steps, chemiluminescent signals were developed using an HRP substrate and visualized using a digital imaging system (iBright 750, Invitrogen, Thermo Fisher Scientific, USA).

### Data analysis and visualization

All data processing and graphical visualization were performed in R (version 4.2.2; R Core Team, 2022). Data handling and summary statistics were generated using the tidyverse package, and figures were created using ggplot2. Growth and pDNA production data are presented as mean values ± standard deviation (SD) of three biological replicates (*n* = 3).

Specific growth rates (µmax) were determined from the slope of linear regressions of ln-transformed OD_600_ values during the exponential growth phase. The exponential phase was identified based on linearity of ln-transformed growth data and defined separately for MSM due to its extended lag phase. Doubling times (DT) were calculated as ln(2)/µ and are reported in minutes.

## Results

The *V. natriegens* Vmax strain employed in this study carries a deletion of dns, which encodes an extracellular, nonspecific endonuclease. This modification parallels the classical endA⁻ strategy implemented in *E. coli strains*. The resulting decrease in nuclease activity limits plasmid degradation during cell disruption and subsequent downstream operations, thereby enhancing the strain’s suitability for pDNA production [27]. For comparative evaluation, *E. coli* K12 DH5α, a standard cloning strain optimized for plasmid stability and transformation efficiency, was used as a reference host [26].

### Growth performance of *Vibrio natriegens* and *Escherichia col*i in different media

**Fig. 1:**
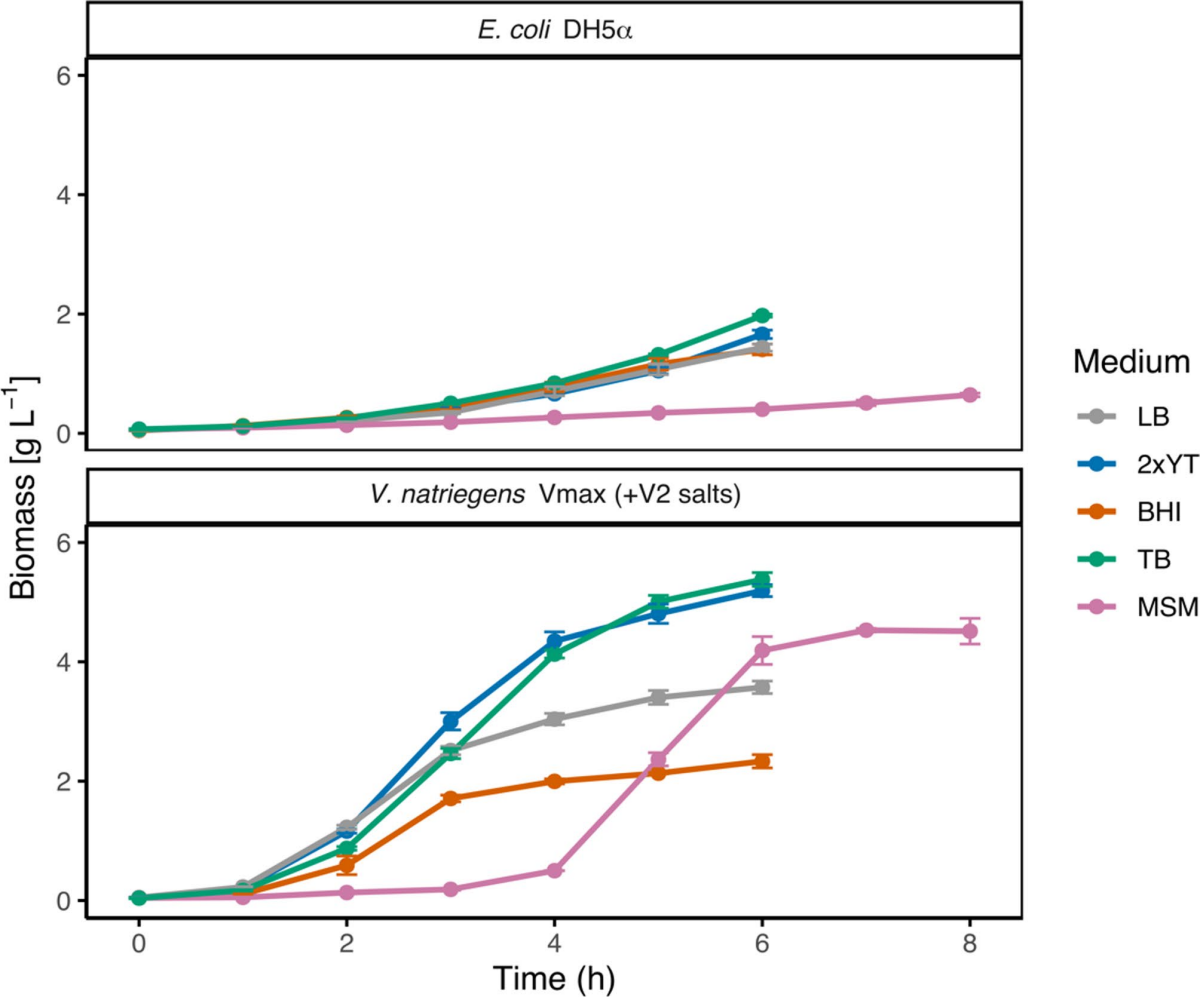
Growth profiles of **A ***V. natriegens* Vmax and **B ***E. coli* DH5α carrying the plasmid peGFP in shake flask cultivations. Biomass concentration is shown as dry cell weight (DCW) over time for four complex media (LB, BHI, 2xYT, TB) and one defined mineral salt medium (MSM). Medium used for *V. natriegens* Vmax was supplemented with V2 salts. Cultivations were performed in triplicates. Data points represent mean values, and error bars indicate standard deviation (SD)

The growth behavior of *Vibrio natriegens* Vmax and *Escherichia coli* DH5α carrying the plasmid peGFP was evaluated in shake flask cultivations using four complex media (LB, BHI, 2xYT, TB) and one defined mineral salt medium (MSM). Biomass formation was quantified as dry cell weight (DCW) over time based on experimentally determined OD_600_-to-DCW conversion factors (Fig. 1).

Across all complex media, *V. natriegens* Vmax exhibited rapid biomass accumulation, reaching high cell densities within 6 h of cultivation (Fig. 1A). In TB and 2xYT, final biomass concentrations of 5.38 ± 0.12 g L⁻¹ and 5.19 ± 0.10 g L⁻¹, respectively, were achieved. Growth in LB resulted in slightly lower final biomass levels (3.57 ± 0.10 g L⁻¹), while BHI supported the lowest biomass formation among the complex media (2.33 ± 0.11 g L⁻¹).

In MSM, *V. natriegens* Vmax showed a pronounced lag phase during the initial cultivation period, followed by a rapid increase in biomass after approximately 4–5 h. After 8 h, DCW values of 4.51 ± 0.22 g L⁻¹ were reached, approaching those observed in complex media despite the chemically defined composition.

In contrast, *E. coli* DH5α displayed substantially slower growth under all tested conditions (Fig. 1B). After 6 h of cultivation in complex media, final biomass concentrations ranged from 1.41 ± 0.09 g L⁻¹ in BHI to 1.97 ± 0.03 g L⁻¹ in TB, with intermediate values observed in LB (1.44 ± 0.06 g L⁻¹) and 2xYT (1.66 ± 0.07 g L⁻¹). Growth of *E. coli* DH5α in MSM was markedly reduced, with DCW values remaining below 0.65 g L⁻¹ even after 8 h of cultivation (0.64 ± 0.03 g L⁻¹).

Overall, *V. natriegens* consistently achieved higher biomass concentrations and reached comparable growth stages substantially earlier than *E. coli* across all tested media.

To quantitatively characterize growth dynamics, specific growth rates (µ_max_) and corresponding doubling times (DT) were determined for *V. natriegens* during the exponential phase in each medium (Table 1). Across complex media, µmax values ranged between 1.21 ± 0.03 h⁻¹ (LB) and 1.39 ± 0.06 h⁻¹ (BHI), corresponding to doubling times of approximately 30–34 min. In MSM, a lower µmax of 1.07 ± 0.03 h⁻¹ was observed, resulting in a doubling time of 39 ± 1 min. These kinetic parameters are consistent with the biomass accumulation profiles shown in Fig. 1.

**Table 1:** Exponential growth parameters of V. natriegens Vmax determined in shake flask cultivations

Parameter	LB (+ V2)	2xYT (+ V2)	BHI (+ V2)	TB (+ V2)	MSM (+ V2)
µ_max_ (h^− 1^)	1.21 ± 0.03	1.34 ± 0.03	1.39 ± 0.06	1.34 ± 0.04	1.07 ± 0.03
DT (min)	34 ± 1	31 ± 1	30 ± 1	31 ± 1	39 ± 1

### Time-resolved plasmid DNA production in shake flask cultures

**Fig. 2:**
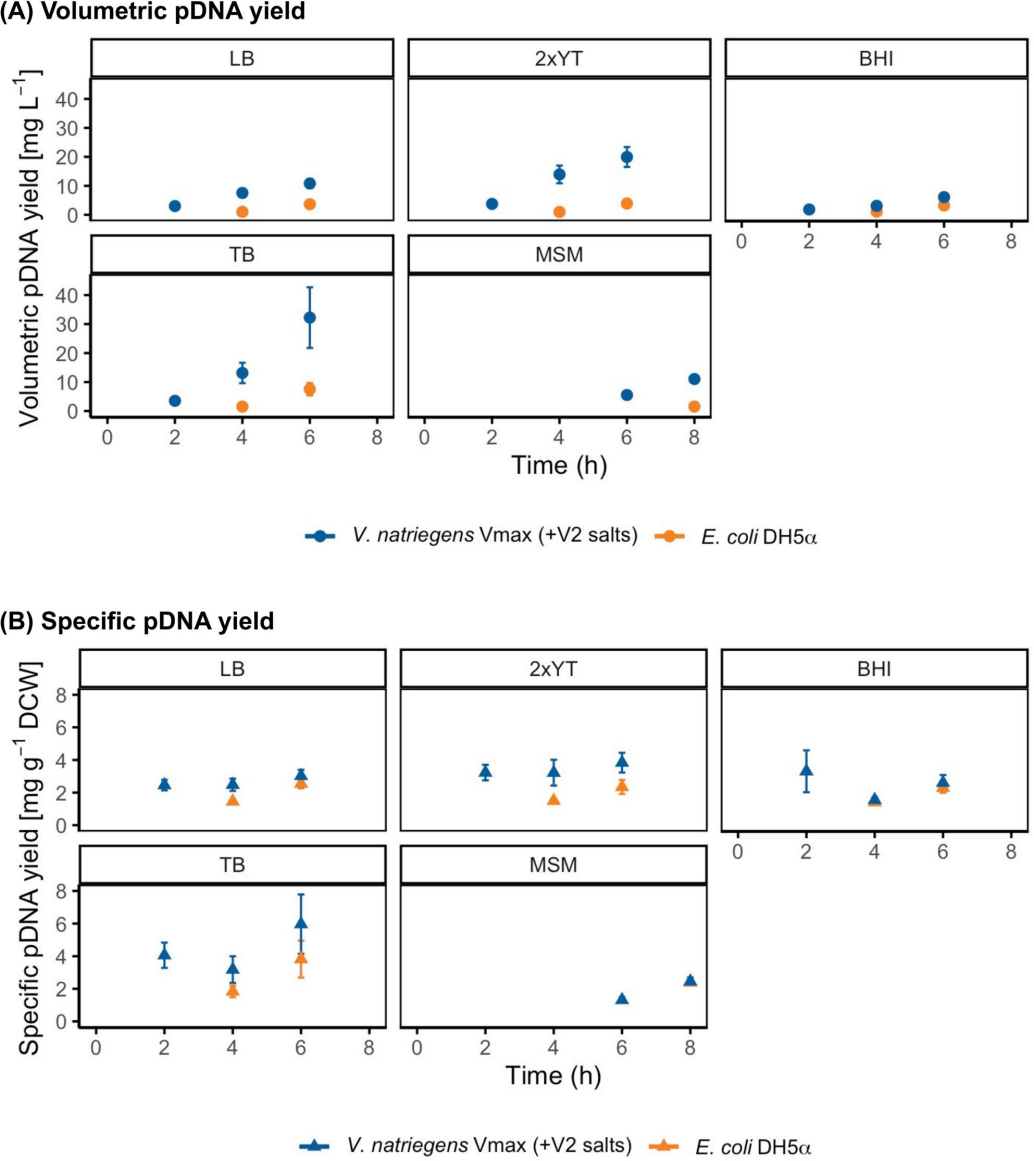
Time-resolved volumetric plasmid DNA (pDNA) production in shake flask cultivations of *V. natriegens* Vmax and *E. coli* DH5α carrying the plasmid peGFP. **A** Volumetric pDNA yields (mg L⁻¹ culture) and **B** biomass-normalized specific pDNA yields (mg g⁻¹ DCW) are shown for four complex media (LB, BHI, 2xYT, TB) and one defined mineral salt medium (MSM). Data represent mean values of three independent biological replicates; error bars indicate standard deviation (SD)

Time-resolved pDNA production of *V. natriegens* Vmax and *E. coli* DH5α carrying the plasmid peGFP was investigated in shake flask cultivations across four complex media (LB, BHI, 2xYT, TB) and one defined mineral salt medium (MSM). Volumetric pDNA yields were determined at selected time points during cultivation and are summarized in Fig. 2A.

In all tested complex media, *V. natriegens* Vmax exhibited earlier onset of detectable pDNA accumulation compared to *E. coli* DH5α (Fig. 2). In 2xYT medium, pDNA was already detected after 2 h of cultivation in *V. natriegens* Vmax cultures (3.76 ± 0.66 mg L⁻¹), whereas no detectable pDNA was observed for *E. coli* DH5α at this time point. pDNA yields in *V. natriegens* Vmax further increased to 13.90 ± 3.05 mg L⁻¹ after 4 h and reached 20.00 ± 3.45 mg L⁻¹ after 6 h. In contrast, *E. coli* DH5α reached only 1.00 ± 0.09 mg L⁻¹ after 4 h and 3.91 ± 0.86 mg L⁻¹ after 6 h.

Similar trends were observed in TB medium, which supported the highest overall pDNA production. *V. natriegens* Vmax achieved 13.10 ± 3.52 mg L⁻¹ after 4 h and 32.20 ± 10.50 mg L⁻¹ after 6 h, whereas *E. coli* DH5α reached 1.54 ± 0.29 mg L⁻¹ and 7.52 ± 2.14 mg L⁻¹ at the respective time points. In LB and BHI media, pDNA accumulation followed the same strain-dependent pattern, with *V. natriegens* Vmax consistently producing higher volumetric yields than *E. coli* DH5α at all comparable sampling times.

In MSM, pDNA production was delayed in both organisms. *V. natriegens* Vmax showed detectable pDNA accumulation after 6 h (5.53 ± 1.03 mg L⁻¹), which increased to 11.00 ± 0.59 mg L⁻¹ after 8 h. In contrast, *E. coli* DH5α produced measurable pDNA only after 8 h of cultivation, reaching 1.54 ± 0.04 mg L⁻¹.

Overall, *V. natriegens* Vmax achieved substantially higher volumetric pDNA yields within shorter cultivation times across all tested media, with particularly pronounced differences observed in nutrient-rich complex media.

### Biomass-normalized specific pDNA yields in shake flask cultures

Biomass-normalized specific pDNA yields of *V. natriegens* Vmax and *E. coli* DH5α carrying the plasmid peGFP were determined by normalizing volumetric pDNA concentrations to biomass concentrations and are summarized in Fig. 2B.

In 2xYT medium, *V. natriegens* Vmax exhibited higher specific pDNA yields at all comparable time points. Specific yields of 3.23 ± 0.48 mg g⁻¹ were already observed after 2 h of cultivation and remained at similar levels after 4 h (3.22 ± 0.79 mg g⁻¹), further increasing to 3.84 ± 0.61 mg g⁻¹ after 6 h. In contrast, *E. coli* DH5α reached 1.50 ± 0.08 mg g⁻¹ after 4 h and 2.34 ± 0.43 mg g⁻¹ after 6 h.

In TB medium, which supported the highest overall specific pDNA yields, *V. natriegens* Vmax achieved 4.06 ± 0.78 mg g⁻¹ after 2 h and increased to 5.97 ± 1.82 mg g⁻¹ after 6 h. *E. coli* DH5α reached 1.84 ± 0.36 mg g⁻¹ after 4 h and 3.82 ± 1.13 mg g⁻¹ after 6 h.

In LB medium, specific pDNA yields of *V. natriegens* Vmax ranged from 2.46 ± 0.32 mg g⁻¹ after 2 h to 3.02 ± 0.38 mg g⁻¹ after 6 h, whereas *E. coli* DH5α reached 1.46 ± 0.11 mg g⁻¹ and 2.55 ± 0.28 mg g⁻¹ at the respective time points. In BHI medium, *V. natriegens* Vmax exhibited 3.31 ± 1.28 mg g⁻¹ after 2 h, followed by 1.55 ± 0.14 mg g⁻¹ after 4 h and 2.60 ± 0.48 mg g⁻¹ after 6 h, while *E. coli* DH5α reached 1.41 ± 0.10 mg g⁻¹ and 2.29 ± 0.30 mg g⁻¹ after 4 h and 6 h, respectively.

In MSM, specific pDNA yields were lower and delayed in both organisms. *V. natriegens* Vmax showed 1.31 ± 0.17 mg g⁻¹ after 6 h and 2.45 ± 0.23 mg g⁻¹ after 8 h, whereas *E. coli* DH5α reached 2.39 ± 0.11 mg g⁻¹ only after 8 h of cultivation.

Overall, *V. natriegens* Vmax exhibited moderately higher specific pDNA yields than *E. coli* DH5α across most media and time points, although differences were less pronounced than those observed for volumetric yields.

### Plasmid DNA quality and functional validation

To assess the structural integrity and functional quality of the pDNA produced in shake flask cultivations, pDNA isolated from *V. natriegens* Vmax and *E. coli* DH5α was analyzed by agarose gel electrophoresis and subsequently evaluated using a cell-free protein expression system.

**Fig. 3:**
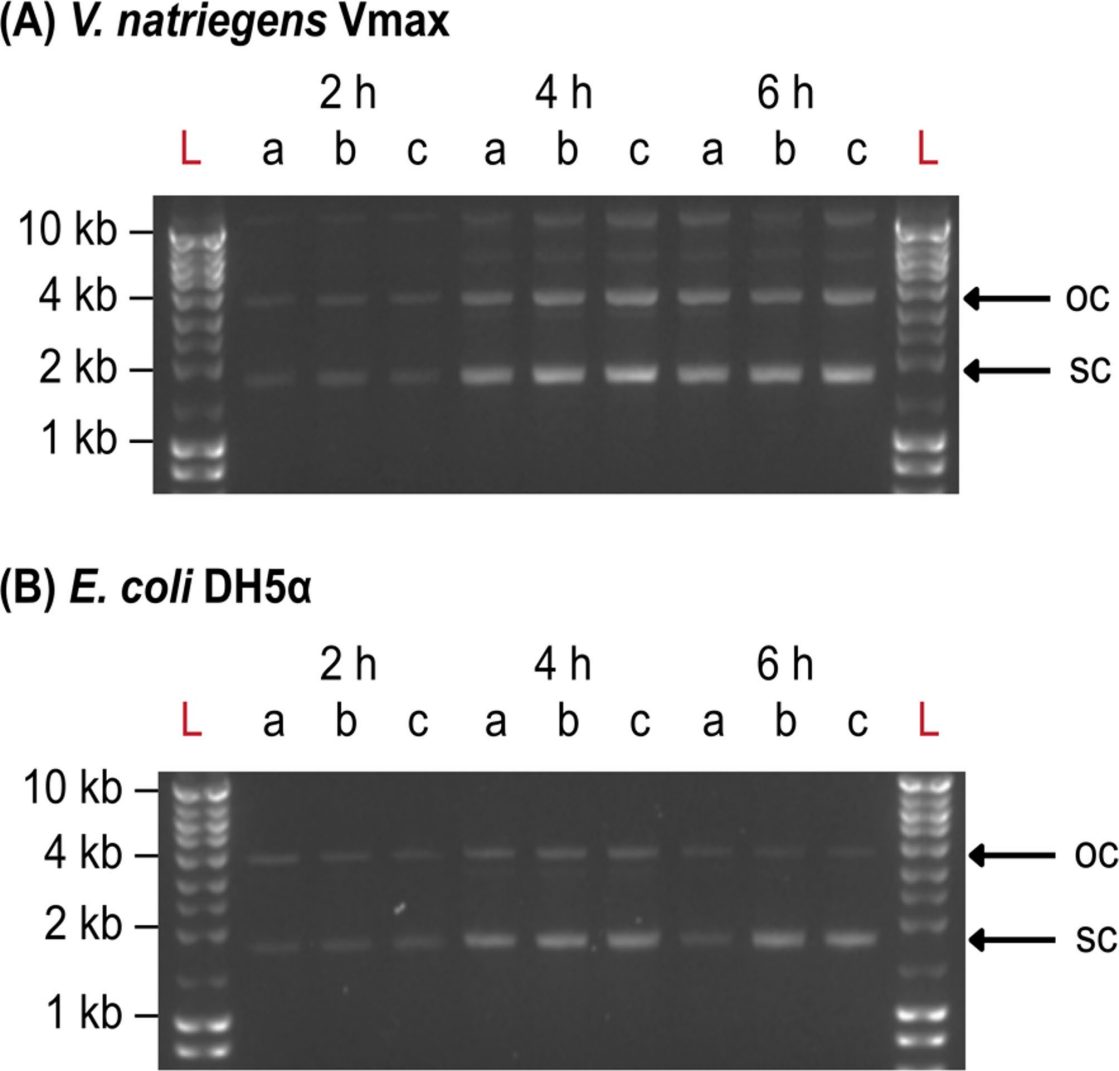
Plasmid DNA (pDNA) topology of peGFP isolated from **A ***V. natriegens* Vmax and **B ***E. coli* DH5α. Agarose gel electrophoresis (1% agarose, 1× TAE) of plasmid DNA (peGFP) isolated after 2, 4, and 6 h of shake-flask cultivation in 2xYT medium (+ V2 for *V. natriegens* Vmax). Left panel: peGFP isolated from *V. natriegens* Vmax. Right panel: peGFP isolated from *E. coli* DH5α. L, NZYDNA Ladder III (200–10,000 bp). Lanes a–c represent biological replicates

Agarose gel analysis confirmed the presence of pDNA in all samples isolated from both host organisms (Fig. 3). For *E. coli* DH5α, the supercoiled pDNA isoform predominated and increased in band intensity over cultivation time. In contrast, pDNA isolated from *V. natriegens* Vmax appeared to exhibit a higher proportion of open circular isoforms and additional slower-migrating topological forms based on agarose gel electrophoresis. This host-specific isoform distribution was consistently observed across biological replicates, sampling times, and cultivation media, including both complex media and the defined minimal medium MSM (Supplementary Fig. S3). However, it should be noted that this assessment is based on qualitative evaluation of gel electrophoresis patterns, and no quantitative analysis of plasmid isoform distribution was performed.

To determine whether the observed pDNA production performance of *V. natriegens* Vmax was limited to a single vector backbone, three additional commonly used high-copy plasmids with different sizes and replication origins were selected. Specifically, pUC18 (~ 2.7 kb, pMB1 origin) and pUC57 (~ 3.9 kb, pMB1 origin) were included as representative small, high-copy-number cloning vectors, while pCEP4 (~ 10 kb, Epstein–Barr virus–based replicon) was chosen to represent a larger and more complex plasmid. This selection enabled assessment of plasmid propagation across different vector architectures and sizes. All plasmids were successfully introduced into *V. natriegens* Vmax and cultivated in 2xYT(+ V2) medium under identical shake flask conditions.

**Fig. 4:**
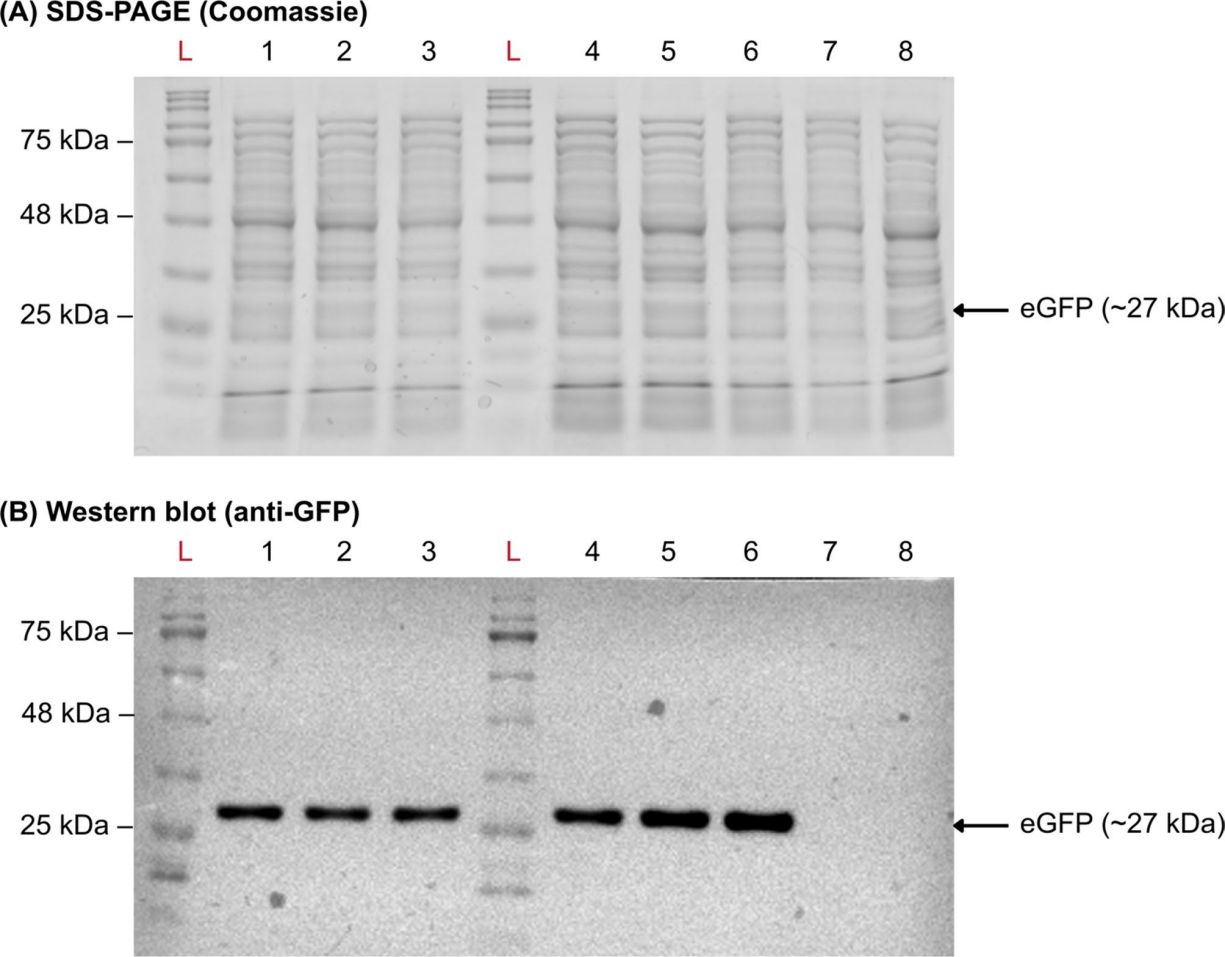
Functional validation of plasmid DNA produced by *V. natriegens* Vmax and *E. coli* DH5α. Plasmid DNA isolated from 6 h shake flask cultures in 2xYT(+ V2) medium was used as template in a cell-free protein expression system for eGFP production. **A** SDS-PAGE analysis of cell-free expression reactions and **B** Western blot detection using an anti-GFP antibody. Lanes 1–3, plasmid DNA isolated from *V. natriegens* Vmax; lanes 4–6, plasmid DNA isolated from *E. coli *DH5α; lane 7, no-template control; lane 8, negative protein control

Growth analysis revealed highly comparable biomass accumulation across all tested plasmids (Fig. 4A), indicating that plasmid carriage did not substantially affect growth behavior. Agarose gel electrophoresis of plasmid preparations obtained after 6 h cultivation confirmed the presence of intact plasmid DNA for all constructs (Fig. 4B). Together, these results demonstrate that efficient plasmid propagation in *V. natriegens* Vmax is not restricted to a single vector backbone, but is compatible with multiple commonly used plasmid types.

**Fig. 5:**
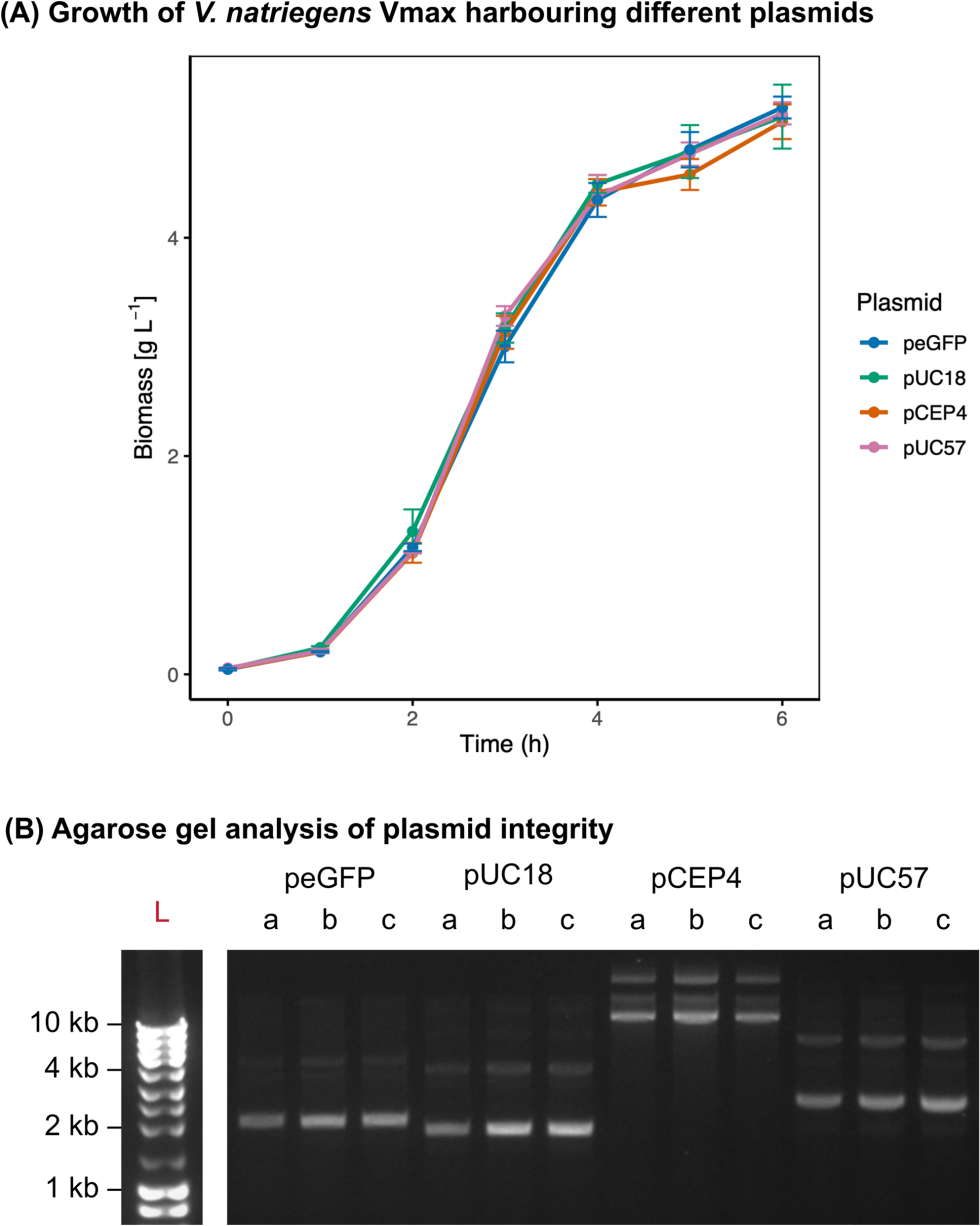
Growth behavior and plasmid integrity of *V. natriegens* Vmax carrying different plasmids. **A** Biomass formation of *V. natriegens* Vmax harboring the plasmids peGFP, pCEP4, pUC18 and pUC57 during shake flask cultivation in 2xYT (+ V2) medium. Biomass was quantified as dry cell weight (DCW) based on OD_600_ conversion factors. Values represent mean ± SD of three biological replicates. **B** Agarose gel electrophoresis of plasmid DNA isolated after 6 h cultivation confirming plasmid integrity for all tested constructs. L: DNA ladder; lanes a–c represent biological replicates

To evaluate whether these differences in pDNA topology affected biological functionality, isolated pDNA was used as template for cell-free expression of enhanced green fluorescent protein (eGFP). Protein production was analyzed by SDS-PAGE and Western blotting using an anti-GFP antibody (Fig. 5). While no distinct eGFP band was detectable by Coomassie staining in the SDS-PAGE, Western blot analysis revealed bands at approximately 27 kDa for all pDNA samples derived from both *V. natriegens* Vmax and *E. coli* DH5α. No signal was detected in the no-template control or in the negative protein control.

These results demonstrate that pDNA produced by *V. natriegens* Vmax is transcriptionally and translationally competent despite differences in plasmid topology. Functional eGFP expression was observed in cell-free transcription–translation reactions using plasmid DNA isolated from both hosts. While fluorescence intensities were somewhat higher for plasmids derived from *E. coli* DH5α, both samples exhibited the characteristic emission maximum of eGFP at approximately 510 nm (Supplementary Fig. S6). Quantification of fluorescence intensity at 510 nm further confirmed measurable protein expression from plasmids isolated from both hosts (Supplementary Tab. S5).

## Discussion

This study provides a proof-of-concept evaluation of *V. natriegens* as an alternative microbial host for plasmid DNA (pDNA) production. By directly comparing *V. natriegens* Vmax and *E. coli* DH5α under identical shake flask conditions across commonly used complex and a defined media, we demonstrate that *V. natriegens* combines exceptionally rapid biomass formation with early and robust plasmid accumulation. Importantly, plasmids isolated from *V. natriegens* were shown to be structurally intact and fully functional in a cell-free expression system, underscoring the suitability of this host for producing biologically active pDNA.

A defining feature of *V. natriegens* observed in this study is its markedly accelerated growth across all tested media. This observation is supported by the experimentally determined specific growth rates, which ranged between 1.07 and 1.39 h⁻¹ across media, corresponding to doubling times of approximately 30–40 min under the tested shake flask conditions. In contrast, *E. coli* exhibited substantially slower biomass accumulation and did not reach comparable cell densities within the same cultivation period (Fig. 1). Independent of nutrient composition, *V. natriegens* consistently reached substantially higher biomass concentrations within shorter cultivation times than *E. coli* DH5α. This kinetic advantage translated directly into earlier availability of pDNA, enabling detectable plasmid recovery at time points where *E. coli* DH5α cultures had not yet reached sufficient cell density for extraction. From a bioprocessing perspective, this temporal shift is highly relevant, as pDNA manufacturing is often constrained by long cultivation times required to accumulate sufficient biomass [2, 5]. Even without strain engineering or process optimization, the rapid growth of *V. natriegens* therefore suggests a clear potential advantage in space–time yield and overall process throughput.

Volumetric pDNA yields reflected this growth-associated benefit. Across all complex media, *V. natriegens* consistently achieved higher pDNA titers within 6 h of cultivation than *E. coli* DH5α, which required longer cultivation times to reach substantially lower yields. When normalized to biomass, specific pDNA yields were generally comparable between *V. natriegens* and *E. coli*, indicating that the higher volumetric yields observed for *V. natriegens* are primarily driven by its rapid biomass accumulation rather than substantially increased intrinsic cellular productivity. These observations are particularly notable given that DH5α represents a standard cloning strain rather than an industrial production host [26]. While optimized *E. coli* production strains can achieve substantially higher absolute titers under fed-batch and induction-based conditions [9, 30], the present comparison under identical batch conditions highlights the intrinsic capacity of *V. natriegens* to rapidly generate pDNA. The data thus extend previous reports on the exceptional growth kinetics of *V. natriegens* [16, 18] by demonstrating that these properties can be directly leveraged for pDNA production.

Medium composition strongly influenced both growth and plasmid production in *V. natriegens*, yet the organism performed robustly across all tested conditions. Nutrient-rich complex media such as TB and 2xYT supported the highest biomass levels and volumetric pDNA yields, consistent with their high availability of amino acids and energy sources. Notably, however, *V. natriegens* also demonstrated effective pDNA production in a chemically defined mineral salt medium. Although cultivation in MSM was associated with a prolonged lag phase, *V. natriegens* ultimately reached biomass concentrations and plasmid titers comparable to those observed in complex media. In contrast, *E. coli* showed severely impaired growth and delayed pDNA production under the same conditions. This indicates that the adaptation to minimal medium is considerably slower in *E. coli* than in *V. natriegens*, highlighting the higher metabolic flexibility of *V. natriegens* under nutrient-limited conditions. The ability of *V. natriegens* to sustain pDNA synthesis in a defined medium is particularly relevant for future process development, as chemically defined formulations offer advantages in reproducibility, controllability, and regulatory compliance [31].

Analysis of plasmid integrity revealed host-dependent differences in pDNA topology. While *E. coli* DH5α predominantly produced supercoiled pDNA, *V. natriegens* Vmax-derived samples appeared to exhibit a higher proportion of open circular and slower-migrating topological forms, which are likely to represent plasmid multimers or concatamers. Restriction analysis confirmed that the isolated plasmids correspond to the expected vector backbones (Supplementary Fig. S4). It should be noted that these observations are based on qualitative assessment of agarose gel electrophoresis patterns, and that no quantitative analysis of plasmid isoform distribution was performed.

These differences may reflect host-specific factors influencing plasmid maintenance and topology. For example, *E. coli* DH5α carries a recombination-deficient (recA⁻) genotype, which is known to limit plasmid recombination [26]. In contrast, no equivalent modification has been reported for the Vmax strain of *V. natriegens*. In addition, differences in growth dynamics and cellular physiology, as discussed above, may contribute to the observed variation in plasmid topology. However, no direct mechanistic link can be established based on the present data. Importantly, the observed isoform distribution was consistent across complex media and the defined MSM, indicating that plasmid topology in *V. natriegens* is primarily host-dependent rather than medium-dependent.

Despite the broader isoform distribution, all *V. natriegens Vmax*-derived plasmids appeared intact and free of degradation, suggesting controlled topological variation rather than structural damage. Similar host-dependent differences in plasmid topology have been reported for alternative non-*E. coli* production systems and do not necessarily compromise downstream applicability [4].

Importantly, the observed growth and plasmid production performance of *V. natriegens* was not limited to a single vector backbone. Comparable growth behavior and successful plasmid recovery were obtained with multiple commonly used high-copy plasmids, indicating that the host supports plasmid propagation across different vector architectures.

Crucially, functional validation demonstrated that pDNA produced in *V. natriegens* is fully competent for gene expression. All plasmid preparations supported robust eGFP synthesis in a cell-free transcription–translation system, as confirmed by Western blot analysis. Specific immunoreactive bands at the expected molecular weight were detected for pDNA derived from both *V. natriegens* and *E. coli*, whereas no signal was observed in the no-template control.

These results demonstrate that plasmids produced in *V. natriegens* are transcriptionally and translationally active despite differences in plasmid topology. The presence of a higher proportion of non-supercoiled isoforms in *V. natriegens*-derived pDNA therefore does not preclude functional gene expression in a heterologous cell-free system. This finding is particularly relevant for research-grade plasmid production and screening applications, where biological functionality is often a more critical criterion than absolute supercoiling content.

Several methodological limitations of this study should be acknowledged that provide opportunities for future research. First, all comparative experiments were conducted at the shake flask level using a single model plasmid for functionality assessment, while only a limited set of additional plasmids was evaluated to assess the general applicability of the host. Furthermore, no strain engineering or plasmid copy-number induction strategies were applied. Second, pDNA quality and topology (e.g., supercoiled versus open circular forms) were evaluated qualitatively by agarose gel electrophoresis, whereas quantitative chromatographic methods were not applied. Third, downstream processing behavior, including lysis efficiency, impurity removal, and purification performance, was not evaluated. In addition, for cultivations in MSM, pre-cultures grown in complex medium (2xYT) were directly used for inoculation without an intermediate washing or adaptation step. This may have resulted in carry-over of residual nutrients, potentially influencing early growth kinetics and reducing the apparent lag phase. Therefore, growth behavior observed in MSM, particularly during the initial cultivation phase, should be interpreted with caution. Finally, comparison to *E. coli* was restricted to a the standard cloning strain DH5α and does not encompass optimized large-scale production hosts. As a result, the performance differences observed may overestimate the relative advantages of *V. natriegens* compared to established high-performance *E. coli* production strains.

It should also be noted that all plasmids used in this study carry an ampicillin resistance marker, which is commonly used in laboratory settings but is generally discouraged for biopharmaceutical applications due to regulatory and stability considerations [6]. Alternative selection systems, such as kanamycin resistance or antibiotic-free approaches, are typically preferred. Kanamycin-based selection systems have also been applied in Gram-negative hosts, including *V. natriegens* [14, 15], although optimization of selection conditions may be required depending on the specific strain and application.

These limitations reflect the intentionally proof-of-concept nature of the present work and define clear directions for future studies.

In summary, this study demonstrates the potential of *V. natriegens* as an alternative host for plasmid DNA production. Its combination of accelerated growth, robust volumetric pDNA yields across diverse media, and the production of functionally competent plasmids highlights its suitability for further exploration in pDNA-related applications. While *V. natriegens* has not yet undergone the extensive genetic and process optimization applied to *E. coli*, the results presented here indicate that efficient pDNA production can be achieved under non-optimized laboratory conditions. Further work focusing on strain engineering (e.g. to reduce levels of homologous recombination), quantitative control of plasmid topology, downstream processing performance, and controlled bioreactor cultivation will be required to comprehensively assess its applicability for large-scale and GMP-compliant production. Nevertheless, the data presented provide a strong experimental basis and support continued investigation of *V. natriegens* as a candidate host for plasmid DNA manufacturing.

## Conclusions

In this study, *V. natriegens* was systematically evaluated as an alternative microbial host for plasmid DNA production and benchmarked against *E. coli* under comparable cultivation conditions. The results demonstrate that *V. natriegens* can produce plasmid DNA of comparable functional performance and apparent integrity based on the analyses performed, while exhibiting distinct growth and production characteristics depending on the applied medium and cultivation strategy.

The combination of time-resolved pDNA quantification, qualitative assessment, and functional validation highlights the potential of *V. natriegens* as a promising platform for pDNA production at laboratory scale. In particular, its rapid growth and early plasmid availability may offer advantages in time-sensitive applications such as cloning, screening, and prototyping workflows.

While large-scale pDNA production typically relies on optimized fed-batch processes and specialized host strains, the present results represent a proof-of-concept under batch conditions and position *V. natriegens* as a complementary system rather than a direct replacement for established *E. coli* production platforms. Further optimization and scale-up studies will be required to assess its full potential in biotechnological applications.

## Supplementary Information

Below is the link to the electronic supplementary material.

Supplementary Material 1.

## Data Availability

All data generated or analysed during this study are included in this published article and its supplementary information files.
