# Supplementary material for: Evaluation of Vibrio natriegens as a fast-growing alternative host for plasmid DNA production
Source: Microb Cell Fact. 2026 May 15;25:156. doi: 10.1186/s12934-026-03026-6 (PMC13343645; doi:10.1186/s12934-026-03026-6)
Supplement: Supplementary file 1 — Supplementary Material 1. [file 12934_2026_3026_MOESM1_ESM.docx]

Supplementary Material:

**Evaluation of *Vibrio natriegens* as a fast-growing alternative host for plasmid DNA production**

Lara S. Möller^1,2,3^, Gabriel A. Monteiro^1,2^, Duarte M.F. Prazeres^1,2^, A. Rita Silva-Santos^1,2^

^1^ iBB – Institute for Bioengineering and Biosciences, Department of Bioengineering, Instituto Superior Técnico, Universidade de Lisboa, Av. Rovisco Pais 1, 1049-001 Lisbon, Portugal

^2^ Associate Laboratory i4HB – Institute for Health and Bioeconomy, Instituto Superior Técnico, Universidade de Lisboa, 1049-001 Lisboa, Portugal

^3^ Hochschule Bremen City University of Applied Sciences, Neustadtswall 30, 28199 Bremen, Germany

Corresponding author:

A. Rita Silva-Santos

ana.rita.santos@tecnico.ulisboa.pt

**
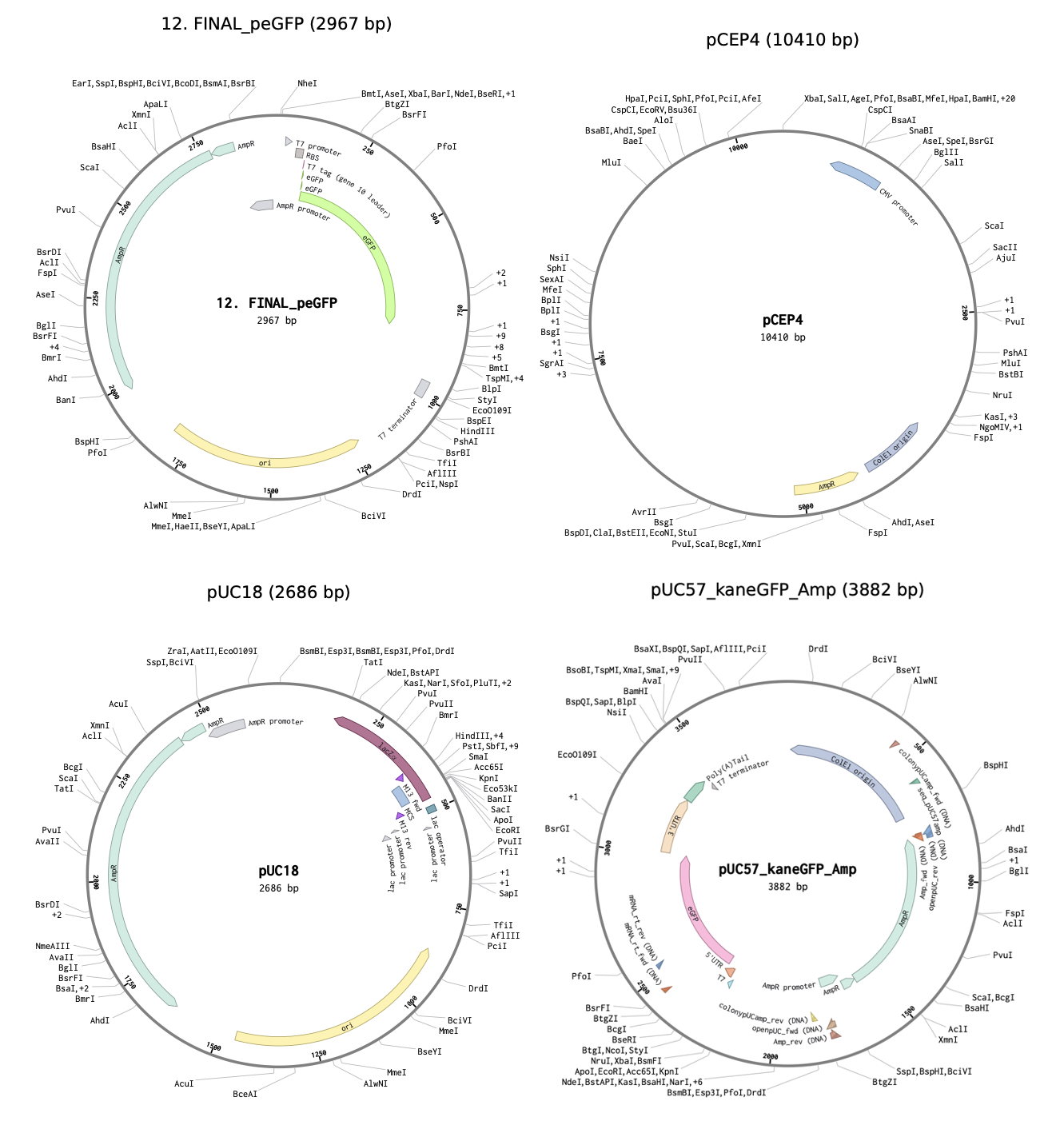
**

***Supplementary Figure S1. Plasmid maps of all vectors used in this study.***

*The figure shows the maps of peGFP (2967 bp), pCEP4 (10,410 bp), pUC18 (2686 bp), and pUC57_kaneGFP_Amp (3882 bp). The peGFP plasmid contains an eGFP expression cassette under control of a T7 promoter, including a ribosome binding site (RBS) and a T terminator. All plasmids carry an ampicillin resistance gene (AmpR) and a ColE1-type origin of replication, unless otherwise indicated.*

***Supplementary Table S2.*** *Composition of culture media used in this study, including medium type, components, manufacturers, and references.*

| **Medium** | **Type** | **Components, Manufacturers and References*** |
| --- | --- | --- |
| LB  (Luria-Bertani) | Complex | LB medium powder (NYZtech, Portugal), prepared according to the manufacturer’s instructions. |
| TB  (Terrific Broth) | Complex | Prepared in-house according to standard formulations as described by Merck (2020):   - Peptone 140 (12 g L⁻¹) - Yeast Extract (24 g L⁻¹) - K_2_HPO_4_ (9.4 g L⁻¹) - KH_2_PO_4_ (2.2 g L⁻¹) - Glycerol (4 mL L⁻¹) |
| 2xYT  (Yeast extract-tryptone) | Complex | 2xYT Broth, granulated medium (Fisher BioReagents, USA), prepared according to the manufacturer’s instructions. |
| BHI  (Brain Heart Infusion) | Complex | Brain Heart Infusion Broth (Merck, USA), prepared according to the manufacturer’s instructions. |
| MSM  (Minimal Salt Medium) | Defined | Prepared in-house according to Soini, Ukkonen and Neubauer, 2008:  *Main salt solution*   - Na_2_SO_4_ (2.0 g L⁻¹) - (NH_4_)_2_SO_4_ (2.47 g L⁻¹) - NH_4_Cl (0.5 g L⁻¹) - K_2_HPO_4_ (14.6 g L⁻¹) - NaH_2_PO_4_·2H_2_O (3.6 g L⁻¹) - (NH_4_)_2_-H-citrate (1.0 g L⁻¹)   ***After heat sterilization***   - MgSO_4_ (2 mL L⁻¹ of 1 M stock solution) - Trace element solution (2 mL L⁻¹, according to Holme et al.,1970)   ***Carbon source***   - D(+)-glucose (10 g L⁻¹, added from stock solution) |
| V2 Salts | Supplement | Prepared in-house according to Weinstock et al., 2016:   - NaCl (204 mM) - KCl (4.2 mM) - MgCl_2_ (23.14 mM) |

*Unless otherwise stated, all chemicals were purchased from Merck (Sigma-Aldrich). Commercial media were prepared according to the manufacturers’ instructions. TB, MSM, and salt solutions were prepared in-house. In experiments using *V. natriegens Vmax*, culture media were supplemented with V2 salts.

***Supplementary Figure S3.*** *Representative agarose gel electrophoresis of plasmid DNA (peGFP) isolated from* V. natriegens*Vmax and* E. coli*DH5α after 6 h and 8 h cultivation in MSM medium (V2 supplementation for* V. natriegens*Vmax). Lanes a–c represent biological replicates. L: NZYDNA Ladder III (200–10,000 bp).*

**

***Supplementary Figure S4****. Agarose gel electrophoresis of plasmid vectors used in this study. The plasmids pUC57, pUC18, peGFP, and pCEP4 were analyzed as uncut plasmid DNA (a) and after restriction digestion (b) to obtain linearized DNA fragments. The expected plasmid sizes are 3882 bp (pUC57), 2686 bp (pUC18), 2967 bp (peGFP), and 10,410 bp (pCEP4). L: DNA ladder.*

***Supplementary Table S5.*** *Fluorescence intensity of eGFP produced in cell-free protein synthesis reactions using plasmid DNA (p-eGFP) isolated from* V. natriegens*Vmax and* E. coli*DH5α. Fluorescence was measured at the emission maximum of 510 nm after excitation at 485 nm. Values represent emission intensity corrected by subtraction of the negative control (no pDNA) and are reported as mean ± standard deviation of three biological replicates (a–c).*

| Strain | A | B | C | Mean ± SD |
| --- | --- | --- | --- | --- |
| V. natriegens Vmax | 1204274 | 2635524 | 1527794 | 1.79 × 10^6^ ± 0.73 × 10^6^ |
| E. coli DH5α | 2842564 | 2507044 | 4028884 | 3.13 × 10^6^ ± 0.78 × 10^6^ |

**

***Supplementary Figure S6.*** *Fluorescence emission spectra of eGFP produced in cell-free protein synthesis reactions using plasmid DNA (peGFP) isolated from* V. natriegens*Vmax (red) and* E. coli *DH5α (blue). Fluorescence emission was recorded from 480 to 680 nm with excitation at 485 nm. Each curve represents an individual biological replicate (n = 3 per strain). The emission maximum was observed at approximately 510 nm, consistent with the characteristic emission peak of eGFP.*

***Supplementary Table S7.*** *Raw experimental data for growth and plasmid DNA production across all strains, media, and plasmids tested in this study. The table includes measured optical density (OD_600_), calculated biomass concentration (g DCW L⁻¹), plasmid DNA concentration in the elution fraction (ng µL⁻¹), purity ratios (A260/A280 and A260/A230), and calculated volumetric pDNA yield (mg L⁻¹) for each biological replicate.*

| Strain | Medium | Plasmid | Replicate | Time_h | OD600_measured | Biomass_DCW_g_L | pDNA_ng_uL_elution | R260_280 | R260_230 | pDNA_mg_L |
| --- | --- | --- | --- | --- | --- | --- | --- | --- | --- | --- |
| Vmax | BHI(+V2) | peGFP | A | 0 | 0.11 | 0.05 |  |  |  |  |
| Vmax | BHI(+V2) | peGFP | B | 0 | 0.08 | 0.04 |  |  |  |  |
| Vmax | BHI(+V2) | peGFP | C | 0 | 0.08 | 0.04 |  |  |  |  |
| Vmax | BHI(+V2) | peGFP | A | 1 | 0.20 | 0.09 |  |  |  |  |
| Vmax | BHI(+V2) | peGFP | B | 1 | 0.24 | 0.11 |  |  |  |  |
| Vmax | BHI(+V2) | peGFP | C | 1 | 0.27 | 0.12 |  |  |  |  |
| Vmax | BHI(+V2) | peGFP | A | 2 | 1.10 | 0.50 | 58.30 | 2.05 | 2.21 | 1.60 |
| Vmax | BHI(+V2) | peGFP | B | 2 | 1.10 | 0.50 | 84.50 | 2.02 | 2.14 | 2.32 |
| Vmax | BHI(+V2) | peGFP | C | 2 | 1.70 | 0.77 | 37.60 | 2.04 | 2.01 | 1.60 |
| Vmax | BHI(+V2) | peGFP | A | 3 | 3.66 | 1.65 |  |  |  |  |
| Vmax | BHI(+V2) | peGFP | B | 3 | 3.83 | 1.72 |  |  |  |  |
| Vmax | BHI(+V2) | peGFP | C | 3 | 3.92 | 1.76 |  |  |  |  |
| Vmax | BHI(+V2) | peGFP | A | 4 | 4.33 | 1.95 | 25.00 | 1.99 | 1.58 | 2.71 |
| Vmax | BHI(+V2) | peGFP | B | 4 | 4.52 | 2.03 | 28.70 | 1.97 | 1.57 | 3.24 |
| Vmax | BHI(+V2) | peGFP | C | 4 | 4.47 | 2.01 | 30.00 | 1.96 | 1.64 | 3.35 |
| Vmax | BHI(+V2) | peGFP | A | 5 | 4.71 | 2.12 |  |  |  |  |
| Vmax | BHI(+V2) | peGFP | B | 5 | 4.75 | 2.14 |  |  |  |  |
| Vmax | BHI(+V2) | peGFP | C | 5 | 4.76 | 2.14 |  |  |  |  |
| Vmax | BHI(+V2) | peGFP | A | 6 | 4.95 | 2.23 | 40.90 | 1.94 | 1.54 | 5.06 |
| Vmax | BHI(+V2) | peGFP | B | 6 | 5.45 | 2.45 | 56.60 | 1.94 | 1.59 | 7.71 |
| Vmax | BHI(+V2) | peGFP | C | 6 | 5.16 | 2.32 | 43.00 | 1.91 | 1.47 | 5.55 |
| Vmax | TB(+V2) | peGFP | A | 0 | 0.10 | 0.05 |  |  |  |  |
| Vmax | TB(+V2) | peGFP | B | 0 | 0.07 | 0.03 |  |  |  |  |
| Vmax | TB(+V2) | peGFP | C | 0 | 0.09 | 0.04 |  |  |  |  |
| Vmax | TB(+V2) | peGFP | A | 1 | 0.36 | 0.16 |  |  |  |  |
| Vmax | TB(+V2) | peGFP | B | 1 | 0.39 | 0.18 |  |  |  |  |
| Vmax | TB(+V2) | peGFP | C | 1 | 0.37 | 0.17 |  |  |  |  |
| Vmax | TB(+V2) | peGFP | A | 2 | 2.01 | 0.90 | 57.20 | 1.98 | 2.11 | 2.87 |
| Vmax | TB(+V2) | peGFP | B | 2 | 1.95 | 0.88 | 77.70 | 1.95 | 2.17 | 3.79 |
| Vmax | TB(+V2) | peGFP | C | 2 | 1.86 | 0.84 | 84.50 | 1.92 | 2.16 | 3.93 |
| Vmax | TB(+V2) | peGFP | A | 3 | 5.52 | 2.48 |  |  |  |  |
| Vmax | TB(+V2) | peGFP | B | 3 | 5.26 | 2.37 |  |  |  |  |
| Vmax | TB(+V2) | peGFP | C | 3 | 5.64 | 2.54 |  |  |  |  |
| Vmax | TB(+V2) | peGFP | A | 4 | 9.21 | 4.14 | 43.50 | 1.98 | 2.14 | 10.02 |
| Vmax | TB(+V2) | peGFP | B | 4 | 9.03 | 4.06 | 55.10 | 1.97 | 2.02 | 12.44 |
| Vmax | TB(+V2) | peGFP | C | 4 | 9.32 | 4.19 | 72.80 | 1.96 | 2.12 | 16.96 |
| Vmax | TB(+V2) | peGFP | A | 5 | 11.39 | 5.13 |  |  |  |  |
| Vmax | TB(+V2) | peGFP | B | 5 | 10.98 | 4.94 |  |  |  |  |
| Vmax | TB(+V2) | peGFP | C | 5 | 11.00 | 4.95 |  |  |  |  |
| Vmax | TB(+V2) | peGFP | A | 6 | 11.72 | 5.27 | 73.20 | 1.93 | 1.89 | 21.45 |
| Vmax | TB(+V2) | peGFP | B | 6 | 11.93 | 5.37 | 110.30 | 1.95 | 2.10 | 32.90 |
| Vmax | TB(+V2) | peGFP | C | 6 | 12.23 | 5.50 | 138.50 | 1.96 | 2.10 | 42.35 |
| Vmax | LB(+V2) | peGFP | A | 0 | 0.10 | 0.05 |  |  |  |  |
| Vmax | LB(+V2) | peGFP | B | 0 | 0.12 | 0.05 |  |  |  |  |
| Vmax | LB(+V2) | peGFP | C | 0 | 0.10 | 0.05 |  |  |  |  |
| Vmax | LB(+V2) | peGFP | A | 1 | 0.48 | 0.22 |  |  |  |  |
| Vmax | LB(+V2) | peGFP | B | 1 | 0.52 | 0.23 |  |  |  |  |
| Vmax | LB(+V2) | peGFP | C | 1 | 0.50 | 0.23 |  |  |  |  |
| Vmax | LB(+V2) | peGFP | A | 2 | 2.64 | 1.19 | 41.60 | 2.03 | 2.35 | 2.75 |
| Vmax | LB(+V2) | peGFP | B | 2 | 2.73 | 1.23 | 51.00 | 2.06 | 2.36 | 3.48 |
| Vmax | LB(+V2) | peGFP | C | 2 | 2.79 | 1.26 | 40.50 | 2.05 | 2.29 | 2.82 |
| Vmax | LB(+V2) | peGFP | A | 3 | 5.76 | 2.59 |  |  |  |  |
| Vmax | LB(+V2) | peGFP | B | 3 | 5.44 | 2.45 |  |  |  |  |
| Vmax | LB(+V2) | peGFP | C | 3 | 5.55 | 2.50 |  |  |  |  |
| Vmax | LB(+V2) | peGFP | A | 4 | 6.51 | 2.93 | 39.80 | 1.96 | 1.82 | 6.48 |
| Vmax | LB(+V2) | peGFP | B | 4 | 6.92 | 3.11 | 41.60 | 2.01 | 1.90 | 7.20 |
| Vmax | LB(+V2) | peGFP | C | 4 | 6.85 | 3.08 | 52.30 | 1.95 | 1.75 | 8.96 |
| Vmax | LB(+V2) | peGFP | A | 5 | 7.26 | 3.27 |  |  |  |  |
| Vmax | LB(+V2) | peGFP | B | 5 | 7.74 | 3.48 |  |  |  |  |
| Vmax | LB(+V2) | peGFP | C | 5 | 7.69 | 3.46 |  |  |  |  |
| Vmax | LB(+V2) | peGFP | A | 6 | 7.76 | 3.49 | 46.60 | 1.96 | 1.94 | 9.04 |
| Vmax | LB(+V2) | peGFP | B | 6 | 7.87 | 3.54 | 59.30 | 1.97 | 2.07 | 11.67 |
| Vmax | LB(+V2) | peGFP | C | 6 | 8.19 | 3.69 | 57.20 | 1.95 | 1.87 | 11.71 |
| Vmax | 2xYT(+V2) | peGFP | A | 0 | 0.09 | 0.04 |  |  |  |  |
| Vmax | 2xYT(+V2) | peGFP | B | 0 | 0.09 | 0.04 |  |  |  |  |
| Vmax | 2xYT(+V2) | peGFP | C | 0 | 0.13 | 0.06 |  |  |  |  |
| Vmax | 2xYT(+V2) | peGFP | A | 1 | 0.47 | 0.21 |  |  |  |  |
| Vmax | 2xYT(+V2) | peGFP | B | 1 | 0.46 | 0.21 |  |  |  |  |
| Vmax | 2xYT(+V2) | peGFP | C | 1 | 0.44 | 0.20 |  |  |  |  |
| Vmax | 2xYT(+V2) | peGFP | A | 2 | 2.52 | 1.13 | 48.40 | 2.03 | 2.19 | 3.05 |
| Vmax | 2xYT(+V2) | peGFP | B | 2 | 2.58 | 1.16 | 60.30 | 2.02 | 2.34 | 3.89 |
| Vmax | 2xYT(+V2) | peGFP | C | 2 | 2.67 | 1.20 | 65.10 | 2.04 | 2.31 | 4.35 |
| Vmax | 2xYT(+V2) | peGFP | A | 3 | 6.50 | 2.93 |  |  |  |  |
| Vmax | 2xYT(+V2) | peGFP | B | 3 | 7.04 | 3.17 |  |  |  |  |
| Vmax | 2xYT(+V2) | peGFP | C | 3 | 6.47 | 2.91 |  |  |  |  |
| Vmax | 2xYT(+V2) | peGFP | A | 4 | 9.80 | 4.41 | 43.50 | 1.97 | 1.99 | 10.66 |
| Vmax | 2xYT(+V2) | peGFP | B | 4 | 9.90 | 4.46 | 58.40 | 1.97 | 2.04 | 14.45 |
| Vmax | 2xYT(+V2) | peGFP | C | 4 | 9.27 | 4.17 | 72.00 | 1.97 | 1.99 | 16.69 |
| Vmax | 2xYT(+V2) | peGFP | A | 5 | 10.27 | 4.62 |  |  |  |  |
| Vmax | 2xYT(+V2) | peGFP | B | 5 | 10.92 | 4.91 |  |  |  |  |
| Vmax | 2xYT(+V2) | peGFP | C | 5 | 10.87 | 4.89 |  |  |  |  |
| Vmax | 2xYT(+V2) | peGFP | A | 6 | 11.29 | 5.08 | 61.90 | 1.93 | 1.92 | 17.47 |
| Vmax | 2xYT(+V2) | peGFP | B | 6 | 11.63 | 5.23 | 63.60 | 1.90 | 1.72 | 18.49 |
| Vmax | 2xYT(+V2) | peGFP | C | 6 | 11.71 | 5.27 | 81.60 | 1.88 | 1.54 | 23.89 |
| Vmax | MSM(+V2) | peGFP | A | 0 | 0.10 | 0.05 |  |  |  |  |
| Vmax | MSM(+V2) | peGFP | B | 0 | 0.10 | 0.05 |  |  |  |  |
| Vmax | MSM(+V2) | peGFP | C | 0 | 0.06 | 0.03 |  |  |  |  |
| Vmax | MSM(+V2) | peGFP | A | 1 | 0.12 | 0.05 |  |  |  |  |
| Vmax | MSM(+V2) | peGFP | B | 1 | 0.12 | 0.05 |  |  |  |  |
| Vmax | MSM(+V2) | peGFP | C | 1 | 0.13 | 0.06 |  |  |  |  |
| Vmax | MSM(+V2) | peGFP | A | 2 | 0.28 | 0.13 |  |  |  |  |
| Vmax | MSM(+V2) | peGFP | B | 2 | 0.30 | 0.14 |  |  |  |  |
| Vmax | MSM(+V2) | peGFP | C | 2 | 0.28 | 0.13 |  |  |  |  |
| Vmax | MSM(+V2) | peGFP | A | 3 | 0.42 | 0.19 |  |  |  |  |
| Vmax | MSM(+V2) | peGFP | B | 3 | 0.45 | 0.20 |  |  |  |  |
| Vmax | MSM(+V2) | peGFP | C | 3 | 0.38 | 0.17 |  |  |  |  |
| Vmax | MSM(+V2) | peGFP | A | 4 | 1.10 | 0.50 |  |  |  |  |
| Vmax | MSM(+V2) | peGFP | B | 4 | 1.11 | 0.50 |  |  |  |  |
| Vmax | MSM(+V2) | peGFP | C | 4 | 1.10 | 0.50 |  |  |  |  |
| Vmax | MSM(+V2) | peGFP | A | 5 | 5.28 | 2.38 |  |  |  |  |
| Vmax | MSM(+V2) | peGFP | B | 5 | 5.48 | 2.47 |  |  |  |  |
| Vmax | MSM(+V2) | peGFP | C | 5 | 5.00 | 2.25 |  |  |  |  |
| Vmax | MSM(+V2) | peGFP | A | 6 | 9.89 | 4.45 | 26.90 | 1.95 | 2.40 | 6.65 |
| Vmax | MSM(+V2) | peGFP | B | 6 | 9.15 | 4.12 | 23.30 | 1.88 | 2.75 | 5.33 |
| Vmax | MSM(+V2) | peGFP | C | 6 | 8.89 | 4.00 | 20.80 | 1.94 | 3.15 | 4.62 |
| Vmax | MSM(+V2) | peGFP | A | 7 | 10.00 | 4.50 |  |  |  |  |
| Vmax | MSM(+V2) | peGFP | B | 7 | 10.13 | 4.56 |  |  |  |  |
| Vmax | MSM(+V2) | peGFP | C | 7 | 10.06 | 4.53 |  |  |  |  |
| Vmax | MSM(+V2) | peGFP | A | 8 | 9.49 | 4.27 | 48.90 | 1.87 | 2.56 | 11.60 |
| Vmax | MSM(+V2) | peGFP | B | 8 | 10.40 | 4.68 | 42.70 | 1.89 | 2.69 | 11.10 |
| Vmax | MSM(+V2) | peGFP | C | 8 | 10.19 | 4.59 | 40.90 | 1.84 | 2.50 | 10.42 |
| DH5 | BHI | peGFP | A | 0 | 0.09 | 0.05 |  |  |  |  |
| DH5 | BHI | peGFP | B | 0 | 0.09 | 0.05 |  |  |  |  |
| DH5 | BHI | peGFP | C | 0 | 0.08 | 0.04 |  |  |  |  |
| DH5 | BHI | peGFP | A | 1 | 0.23 | 0.12 |  |  |  |  |
| DH5 | BHI | peGFP | B | 1 | 0.25 | 0.13 |  |  |  |  |
| DH5 | BHI | peGFP | C | 1 | 0.25 | 0.13 |  |  |  |  |
| DH5 | BHI | peGFP | A | 2 | 0.48 | 0.24 |  |  |  |  |
| DH5 | BHI | peGFP | B | 2 | 0.52 | 0.26 |  |  |  |  |
| DH5 | BHI | peGFP | C | 2 | 0.57 | 0.29 |  |  |  |  |
| DH5 | BHI | peGFP | A | 3 | 0.81 | 0.41 |  |  |  |  |
| DH5 | BHI | peGFP | B | 3 | 0.84 | 0.42 |  |  |  |  |
| DH5 | BHI | peGFP | C | 3 | 0.98 | 0.49 |  |  |  |  |
| DH5 | BHI | peGFP | A | 4 | 1.39 | 0.70 | 28.70 | 2.03 | 1.98 | 1.00 |
| DH5 | BHI | peGFP | B | 4 | 1.54 | 0.77 | 26.10 | 2.04 | 1.90 | 1.00 |
| DH5 | BHI | peGFP | C | 4 | 1.71 | 0.86 | 30.20 | 2.10 | 1.91 | 1.29 |
| DH5 | BHI | peGFP | A | 5 | 2.09 | 1.05 |  |  |  |  |
| DH5 | BHI | peGFP | B | 5 | 2.36 | 1.18 |  |  |  |  |
| DH5 | BHI | peGFP | C | 5 | 2.49 | 1.25 |  |  |  |  |
| DH5 | BHI | peGFP | A | 6 | 2.83 | 1.42 | 41.80 | 1.97 | 1.51 | 2.96 |
| DH5 | BHI | peGFP | B | 6 | 2.62 | 1.31 | 43.10 | 1.92 | 1.33 | 2.82 |
| DH5 | BHI | peGFP | C | 6 | 2.98 | 1.49 | 52.80 | 1.95 | 1.44 | 3.93 |
| DH5 | TB | peGFP | A | 0 | 0.11 | 0.06 |  |  |  |  |
| DH5 | TB | peGFP | B | 0 | 0.15 | 0.08 |  |  |  |  |
| DH5 | TB | peGFP | C | 0 | 0.11 | 0.06 |  |  |  |  |
| DH5 | TB | peGFP | A | 1 | 0.23 | 0.12 |  |  |  |  |
| DH5 | TB | peGFP | B | 1 | 0.23 | 0.12 |  |  |  |  |
| DH5 | TB | peGFP | C | 1 | 0.23 | 0.12 |  |  |  |  |
| DH5 | TB | peGFP | A | 2 | 0.47 | 0.24 |  |  |  |  |
| DH5 | TB | peGFP | B | 2 | 0.52 | 0.26 |  |  |  |  |
| DH5 | TB | peGFP | C | 2 | 0.52 | 0.26 |  |  |  |  |
| DH5 | TB | peGFP | A | 3 | 0.98 | 0.49 |  |  |  |  |
| DH5 | TB | peGFP | B | 3 | 1.03 | 0.52 |  |  |  |  |
| DH5 | TB | peGFP | C | 3 | 1.00 | 0.50 |  |  |  |  |
| DH5 | TB | peGFP | A | 4 | 1.65 | 0.83 | 35.50 | 2.05 | 1.99 | 1.46 |
| DH5 | TB | peGFP | B | 4 | 1.70 | 0.85 | 30.60 | 2.06 | 2.06 | 1.30 |
| DH5 | TB | peGFP | C | 4 | 1.68 | 0.84 | 44.60 | 1.98 | 1.76 | 1.87 |
| DH5 | TB | peGFP | A | 5 | 2.59 | 1.30 |  |  |  |  |
| DH5 | TB | peGFP | B | 5 | 2.64 | 1.32 |  |  |  |  |
| DH5 | TB | peGFP | C | 5 | 2.65 | 1.33 |  |  |  |  |
| DH5 | TB | peGFP | A | 6 | 3.89 | 1.95 | 100.60 | 2.00 | 1.87 | 9.78 |
| DH5 | TB | peGFP | B | 6 | 3.94 | 1.97 | 73.60 | 2.01 | 1.79 | 7.25 |
| DH5 | TB | peGFP | C | 6 | 4.00 | 2.00 | 55.30 | 2.00 | 1.67 | 5.53 |
| DH5 | LB | peGFP | A | 0 | 0.09 | 0.05 |  |  |  |  |
| DH5 | LB | peGFP | B | 0 | 0.11 | 0.06 |  |  |  |  |
| DH5 | LB | peGFP | C | 0 | 0.10 | 0.05 |  |  |  |  |
| DH5 | LB | peGFP | A | 1 | 0.19 | 0.10 |  |  |  |  |
| DH5 | LB | peGFP | B | 1 | 0.21 | 0.11 |  |  |  |  |
| DH5 | LB | peGFP | C | 1 | 0.21 | 0.11 |  |  |  |  |
| DH5 | LB | peGFP | A | 2 | 0.40 | 0.20 |  |  |  |  |
| DH5 | LB | peGFP | B | 2 | 0.41 | 0.21 |  |  |  |  |
| DH5 | LB | peGFP | C | 2 | 0.44 | 0.22 |  |  |  |  |
| DH5 | LB | peGFP | A | 3 | 0.70 | 0.35 |  |  |  |  |
| DH5 | LB | peGFP | B | 3 | 0.74 | 0.37 |  |  |  |  |
| DH5 | LB | peGFP | C | 3 | 0.65 | 0.33 |  |  |  |  |
| DH5 | LB | peGFP | A | 4 | 1.27 | 0.64 | 29.30 | 2.03 | 2.24 | 0.93 |
| DH5 | LB | peGFP | B | 4 | 1.37 | 0.69 | 27.20 | 2.07 | 2.32 | 0.93 |
| DH5 | LB | peGFP | C | 4 | 1.57 | 0.79 | 31.50 | 2.08 | 2.36 | 1.24 |
| DH5 | LB | peGFP | A | 5 | 1.96 | 0.98 |  |  |  |  |
| DH5 | LB | peGFP | B | 5 | 2.15 | 1.08 |  |  |  |  |
| DH5 | LB | peGFP | C | 5 | 2.31 | 1.16 |  |  |  |  |
| DH5 | LB | peGFP | A | 6 | 2.76 | 1.38 | 57.40 | 1.84 | 1.34 | 3.96 |
| DH5 | LB | peGFP | B | 6 | 2.85 | 1.43 | 46.70 | 1.87 | 1.77 | 3.33 |
| DH5 | LB | peGFP | C | 6 | 2.99 | 1.50 | 49.20 | 1.97 | 1.96 | 3.68 |
| DH5 | 2xYT | peGFP | A | 0 | 0.13 | 0.07 |  |  |  |  |
| DH5 | 2xYT | peGFP | B | 0 | 0.13 | 0.07 |  |  |  |  |
| DH5 | 2xYT | peGFP | C | 0 | 0.13 | 0.07 |  |  |  |  |
| DH5 | 2xYT | peGFP | A | 1 | 0.19 | 0.10 |  |  |  |  |
| DH5 | 2xYT | peGFP | B | 1 | 0.20 | 0.10 |  |  |  |  |
| DH5 | 2xYT | peGFP | C | 1 | 0.21 | 0.11 |  |  |  |  |
| DH5 | 2xYT | peGFP | A | 2 | 0.44 | 0.22 |  |  |  |  |
| DH5 | 2xYT | peGFP | B | 2 | 0.44 | 0.22 |  |  |  |  |
| DH5 | 2xYT | peGFP | C | 2 | 0.45 | 0.23 |  |  |  |  |
| DH5 | 2xYT | peGFP | A | 3 | 0.77 | 0.39 |  |  |  |  |
| DH5 | 2xYT | peGFP | B | 3 | 0.80 | 0.40 |  |  |  |  |
| DH5 | 2xYT | peGFP | C | 3 | 0.86 | 0.43 |  |  |  |  |
| DH5 | 2xYT | peGFP | A | 4 | 1.28 | 0.64 | 28.30 | 2.01 | 2.07 | 0.91 |
| DH5 | 2xYT | peGFP | B | 4 | 1.32 | 0.66 | 29.90 | 2.02 | 2.07 | 0.99 |
| DH5 | 2xYT | peGFP | C | 4 | 1.38 | 0.69 | 31.70 | 2.07 | 2.07 | 1.09 |
| DH5 | 2xYT | peGFP | A | 5 | 2.00 | 1.00 |  |  |  |  |
| DH5 | 2xYT | peGFP | B | 5 | 2.08 | 1.04 |  |  |  |  |
| DH5 | 2xYT | peGFP | C | 5 | 2.22 | 1.11 |  |  |  |  |
| DH5 | 2xYT | peGFP | A | 6 | 3.15 | 1.58 | 37.10 | 1.94 | 1.64 | 2.92 |
| DH5 | 2xYT | peGFP | B | 6 | 3.37 | 1.69 | 52.40 | 1.98 | 1.71 | 4.41 |
| DH5 | 2xYT | peGFP | C | 6 | 3.41 | 1.71 | 51.60 | 2.01 | 1.79 | 4.40 |
| DH5 | MSM | peGFP | A | 0 | 0.11 | 0.06 |  |  |  |  |
| DH5 | MSM | peGFP | B | 0 | 0.13 | 0.07 |  |  |  |  |
| DH5 | MSM | peGFP | C | 0 | 0.13 | 0.07 |  |  |  |  |
| DH5 | MSM | peGFP | A | 1 | 0.20 | 0.10 |  |  |  |  |
| DH5 | MSM | peGFP | B | 1 | 0.17 | 0.09 |  |  |  |  |
| DH5 | MSM | peGFP | C | 1 | 0.18 | 0.09 |  |  |  |  |
| DH5 | MSM | peGFP | A | 2 | 0.29 | 0.15 |  |  |  |  |
| DH5 | MSM | peGFP | B | 2 | 0.26 | 0.13 |  |  |  |  |
| DH5 | MSM | peGFP | C | 2 | 0.26 | 0.13 |  |  |  |  |
| DH5 | MSM | peGFP | A | 3 | 0.37 | 0.19 |  |  |  |  |
| DH5 | MSM | peGFP | B | 3 | 0.36 | 0.18 |  |  |  |  |
| DH5 | MSM | peGFP | C | 3 | 0.37 | 0.19 |  |  |  |  |
| DH5 | MSM | peGFP | A | 4 | 0.50 | 0.25 |  |  |  |  |
| DH5 | MSM | peGFP | B | 4 | 0.57 | 0.29 |  |  |  |  |
| DH5 | MSM | peGFP | C | 4 | 0.52 | 0.26 |  |  |  |  |
| DH5 | MSM | peGFP | A | 5 | 0.67 | 0.34 |  |  |  |  |
| DH5 | MSM | peGFP | B | 5 | 0.68 | 0.34 |  |  |  |  |
| DH5 | MSM | peGFP | C | 5 | 0.70 | 0.35 |  |  |  |  |
| DH5 | MSM | peGFP | A | 6 | 0.77 | 0.39 |  |  |  |  |
| DH5 | MSM | peGFP | B | 6 | 0.80 | 0.40 |  |  |  |  |
| DH5 | MSM | peGFP | C | 6 | 0.83 | 0.42 |  |  |  |  |
| DH5 | MSM | peGFP | A | 7 | 0.92 | 0.46 |  |  |  |  |
| DH5 | MSM | peGFP | B | 7 | 1.04 | 0.52 |  |  |  |  |
| DH5 | MSM | peGFP | C | 7 | 1.10 | 0.55 |  |  |  |  |
| DH5 | MSM | peGFP | A | 8 | 1.22 | 0.61 | 49.50 | 1.97 | 3.05 | 1.51 |
| DH5 | MSM | peGFP | B | 8 | 1.29 | 0.65 | 48.90 | 1.92 | 2.47 | 1.58 |
| DH5 | MSM | peGFP | C | 8 | 1.34 | 0.67 | 45.30 | 1.92 | 2.72 | 1.52 |
| Vmax | 2xYT(+V2) | pCEP4 | A | 0 | 0.11 | 0.05 |  |  |  |  |
| Vmax | 2xYT(+V2) | pCEP4 | B | 0 | 0.11 | 0.05 |  |  |  |  |
| Vmax | 2xYT(+V2) | pCEP4 | C | 0 | 0.11 | 0.05 |  |  |  |  |
| Vmax | 2xYT(+V2) | pCEP4 | A | 1 | 0.44 | 0.20 |  |  |  |  |
| Vmax | 2xYT(+V2) | pCEP4 | B | 1 | 0.46 | 0.21 |  |  |  |  |
| Vmax | 2xYT(+V2) | pCEP4 | C | 1 | 0.46 | 0.21 |  |  |  |  |
| Vmax | 2xYT(+V2) | pCEP4 | A | 2 | 2.29 | 1.03 |  |  |  |  |
| Vmax | 2xYT(+V2) | pCEP4 | B | 2 | 2.44 | 1.10 |  |  |  |  |
| Vmax | 2xYT(+V2) | pCEP4 | C | 2 | 2.69 | 1.21 |  |  |  |  |
| Vmax | 2xYT(+V2) | pCEP4 | A | 3 | 6.58 | 2.96 |  |  |  |  |
| Vmax | 2xYT(+V2) | pCEP4 | B | 3 | 7.17 | 3.23 |  |  |  |  |
| Vmax | 2xYT(+V2) | pCEP4 | C | 3 | 7.13 | 3.21 |  |  |  |  |
| Vmax | 2xYT(+V2) | pCEP4 | A | 4 | 9.91 | 4.46 |  |  |  |  |
| Vmax | 2xYT(+V2) | pCEP4 | B | 4 | 10.02 | 4.51 |  |  |  |  |
| Vmax | 2xYT(+V2) | pCEP4 | C | 4 | 9.51 | 4.28 |  |  |  |  |
| Vmax | 2xYT(+V2) | pCEP4 | A | 5 | 10.06 | 4.53 |  |  |  |  |
| Vmax | 2xYT(+V2) | pCEP4 | B | 5 | 10.54 | 4.74 |  |  |  |  |
| Vmax | 2xYT(+V2) | pCEP4 | C | 5 | 9.94 | 4.47 |  |  |  |  |
| Vmax | 2xYT(+V2) | pCEP4 | A | 6 | 11.46 | 5.16 | 38.20 | 1.78 | 1.14 | 10.94 |
| Vmax | 2xYT(+V2) | pCEP4 | B | 6 | 10.84 | 4.88 | 59.20 | 1.82 | 1.32 | 16.04 |
| Vmax | 2xYT(+V2) | pCEP4 | C | 6 | 11.45 | 5.15 | 70.40 | 1.85 | 1.43 | 20.15 |
| Vmax | 2xYT(+V2) | pUC18 | A | 0 | 0.11 | 0.05 |  |  |  |  |
| Vmax | 2xYT(+V2) | pUC18 | B | 0 | 0.12 | 0.05 |  |  |  |  |
| Vmax | 2xYT(+V2) | pUC18 | C | 0 | 0.11 | 0.05 |  |  |  |  |
| Vmax | 2xYT(+V2) | pUC18 | A | 1 | 0.54 | 0.24 |  |  |  |  |
| Vmax | 2xYT(+V2) | pUC18 | B | 1 | 0.52 | 0.23 |  |  |  |  |
| Vmax | 2xYT(+V2) | pUC18 | C | 1 | 0.58 | 0.26 |  |  |  |  |
| Vmax | 2xYT(+V2) | pUC18 | A | 2 | 2.96 | 1.33 |  |  |  |  |
| Vmax | 2xYT(+V2) | pUC18 | B | 2 | 2.45 | 1.10 |  |  |  |  |
| Vmax | 2xYT(+V2) | pUC18 | C | 2 | 3.33 | 1.50 |  |  |  |  |
| Vmax | 2xYT(+V2) | pUC18 | A | 3 | 6.72 | 3.02 |  |  |  |  |
| Vmax | 2xYT(+V2) | pUC18 | B | 3 | 7.26 | 3.27 |  |  |  |  |
| Vmax | 2xYT(+V2) | pUC18 | C | 3 | 7.17 | 3.23 |  |  |  |  |
| Vmax | 2xYT(+V2) | pUC18 | A | 4 | 9.78 | 4.40 |  |  |  |  |
| Vmax | 2xYT(+V2) | pUC18 | B | 4 | 10.07 | 4.53 |  |  |  |  |
| Vmax | 2xYT(+V2) | pUC18 | C | 4 | 10.11 | 4.55 |  |  |  |  |
| Vmax | 2xYT(+V2) | pUC18 | A | 5 | 10.02 | 4.51 |  |  |  |  |
| Vmax | 2xYT(+V2) | pUC18 | B | 5 | 10.98 | 4.94 |  |  |  |  |
| Vmax | 2xYT(+V2) | pUC18 | C | 5 | 10.93 | 4.92 |  |  |  |  |
| Vmax | 2xYT(+V2) | pUC18 | A | 6 | 10.62 | 4.78 | 39.20 | 1.84 | 1.31 | 10.41 |
| Vmax | 2xYT(+V2) | pUC18 | B | 6 | 11.57 | 5.21 | 66.20 | 1.82 | 1.32 | 19.15 |
| Vmax | 2xYT(+V2) | pUC18 | C | 6 | 11.86 | 5.34 | 51.40 | 1.85 | 1.36 | 15.24 |
| Vmax | 2xYT(+V2) | pUC57 | A | 0 | 0.13 | 0.06 |  |  |  |  |
| Vmax | 2xYT(+V2) | pUC57 | B | 0 | 0.13 | 0.06 |  |  |  |  |
| Vmax | 2xYT(+V2) | pUC57 | C | 0 | 0.11 | 0.05 |  |  |  |  |
| Vmax | 2xYT(+V2) | pUC57 | A | 1 | 0.42 | 0.19 |  |  |  |  |
| Vmax | 2xYT(+V2) | pUC57 | B | 1 | 0.49 | 0.22 |  |  |  |  |
| Vmax | 2xYT(+V2) | pUC57 | C | 1 | 0.51 | 0.23 |  |  |  |  |
| Vmax | 2xYT(+V2) | pUC57 | A | 2 | 2.47 | 1.11 |  |  |  |  |
| Vmax | 2xYT(+V2) | pUC57 | B | 2 | 2.50 | 1.13 |  |  |  |  |
| Vmax | 2xYT(+V2) | pUC57 | C | 2 | 2.49 | 1.12 |  |  |  |  |
| Vmax | 2xYT(+V2) | pUC57 | A | 3 | 7.08 | 3.19 |  |  |  |  |
| Vmax | 2xYT(+V2) | pUC57 | B | 3 | 7.30 | 3.29 |  |  |  |  |
| Vmax | 2xYT(+V2) | pUC57 | C | 3 | 7.48 | 3.37 |  |  |  |  |
| Vmax | 2xYT(+V2) | pUC57 | A | 4 | 9.25 | 4.16 |  |  |  |  |
| Vmax | 2xYT(+V2) | pUC57 | B | 4 | 9.97 | 4.49 |  |  |  |  |
| Vmax | 2xYT(+V2) | pUC57 | C | 4 | 10.00 | 4.50 |  |  |  |  |
| Vmax | 2xYT(+V2) | pUC57 | A | 5 | 10.65 | 4.79 |  |  |  |  |
| Vmax | 2xYT(+V2) | pUC57 | B | 5 | 10.34 | 4.65 |  |  |  |  |
| Vmax | 2xYT(+V2) | pUC57 | C | 5 | 10.80 | 4.86 |  |  |  |  |
| Vmax | 2xYT(+V2) | pUC57 | A | 6 | 11.18 | 5.03 | 41.70 | 1.80 | 1.17 | 11.66 |
| Vmax | 2xYT(+V2) | pUC57 | B | 6 | 11.46 | 5.16 | 54.90 | 1.82 | 1.18 | 15.73 |
| Vmax | 2xYT(+V2) | pUC57 | C | 6 | 11.63 | 5.23 | 50.50 | 1.80 | 1.20 | 14.68 |
